# Accurate Kinetic Studies of OH + HO_2_ Radical–Radical Reaction through Direct Measurement of Precursor and Radical Concentrations with High-Resolution Time-Resolved Dual-Comb Spectroscopy

**DOI:** 10.1021/acs.jpclett.4c00494

**Published:** 2024-03-28

**Authors:** I-Yun Chen, Che-Wei Chang, Christa Fittschen, Pei-Ling Luo

**Affiliations:** † Institute of Atomic and Molecular Sciences, Academia Sinica, Taipei 106319, Taiwan; ‡ Department of Chemistry, National Taiwan University, Taipei 10617, Taiwan; § Molecular Science and Technology Program, Taiwan International Graduate Program, Academia Sinica, 11529 Taipei, Taiwan; ∥ International Graduate Program of Molecular Science and Technology, National Taiwan University, 10617 Taipei, Taiwan; ⊥ University Lille, CNRS, UMR 8522, PC2A−Physicochimie des Processus de Combustion et de l’Atmosphère, F-59000 Lille, France

## Abstract

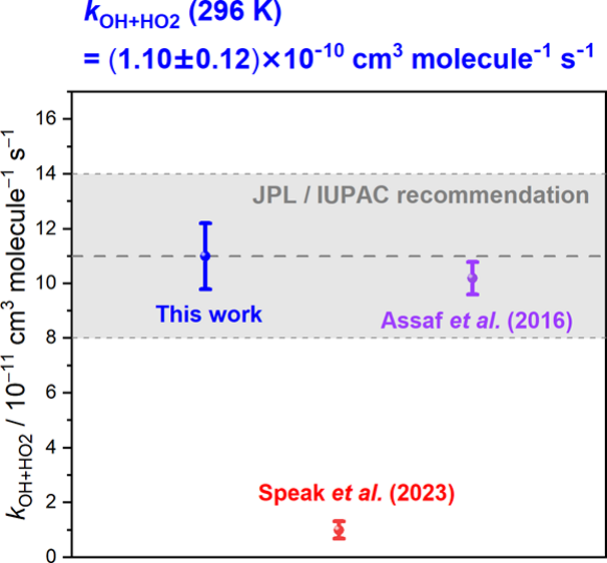

The radical–radical reaction between OH and HO_2_ has been considered for a long time as an important reaction in tropospheric photochemistry and combustion chemistry. However, a significant discrepancy of an order of magnitude for rate coefficients of this reaction is found between two recent experiments. Herein, we investigate the reaction OH + HO_2_ via direct spectral quantification of both the precursor (H_2_O_2_) and free radicals (OH and HO_2_) upon the 248 nm photolysis of H_2_O_2_ using infrared two-color time-resolved dual-comb spectroscopy. With quantitative and kinetic analysis of concentration profiles of both OH and HO_2_ at varied conditions, the rate coefficient *k*_OH+HO_2__ is determined to be (1.10 ± 0.12) × 10^–10^ cm^3^ molecule^–1^ s^–1^ at 296 K. Moreover, we explore the kinetics of this reaction under conditions in the presence of water, but no enhancement in the *k*_OH+HO_2__ can be observed. This work as an independent experiment plays a crucial role in revisiting this prototypical radical–radical reaction.

As atmospheric detergents, hydroxyl (OH) and hydroperoxyl (HO_2_) radicals play critical roles in the catalytic cycle of ozone formation and controlling secondary pollution in the troposphere.^1,2^ The reaction of OH with HO_2_ is a key pathway for removal of both OH and HO_2_ radicals in the upper troposphere, and it is also of special importance in low-NO_*x*_ atmosphere and low-temperature combustion systems.^3−5^ In addition, being a prototype of the radical–radical reaction, the reaction between OH and HO_2_ is also of great interest in both theoretical and physical chemistry. A brief description of the previous studies of the reaction OH + HO_2_ is shown in Note S1. Although numerous investigations on the kinetics of the reaction OH + HO_2_ have been conducted during the past couple of decades,^6−17^ the reported rate coefficients *k*_OH+HO_2__ from both experimental and theoretical studies are scattered in a wide range of (0.1–1.7) × 10^–10^ cm^3^ molecule^–1^ s^–1^ at around room temperature, as shown in Figure S1. In particular, the most recent measurements of the rate coefficient carried out by Assaf et al.^6^ and Speak et al.^7^ show a significant discrepancy between each other by a factor of 10. Assaf et al. employed laser-induced fluorescence (LIF) and near-infrared cavity ring down spectroscopy (CRDS) to respectively monitor the OH and HO_2_ radicals generated upon laser flash photolysis (LFP) of the mixture of H_2_O_2_/(COCl)_2_/CH_3_OH/O_2_ to investigate the kinetics of the reaction OH + HO_2_.^6^ By analyzing the time traces of OH and HO_2_ obtained at different experimental conditions and kinetic simulations with adjustable parameters of initial concentrations of H_2_O_2_ and OH, Assaf et al. obtained the rate coefficient *k*_OH+HO_2__ with a value of (1.02 ± 0.06) × 10^–10^ cm^3^ molecule^–1^ s^–1^ at 298 K. Most recently, Speak et al. conducted kinetic measurements of the reaction OH + HO_2_ in the LFP and LIF systems.^7^ By adopting the multiple reactions such as OH + H_2_O_2_, OH + CH_3_OH, and HO_2_ + NO, Speak et al. calibrated the LIF signals of OH as well as the relative yields of HO_2_ in the reactors. The rate coefficients *k*_OH+HO_2__ at 298 K were obtained with the values of (1.34 ± 0.14) and (1.00 ± 0.32) × 10^–11^ cm^3^ molecule^–1^ s^–1^ from the high-pressure and low-pressure studies, respectively. Furthermore, a significant enhancement in the rate coefficient *k*_OH+HO_2__ in the presence of water was also observed in the high-pressure water photolysis experiments with excessive oxygen by Speak et al. Both experimental studies have been carefully conducted; however, in these experiments, the initial concentrations of the precursors such as hydrogen peroxide (H_2_O_2_) and the radical concentrations were estimated mainly based on earlier kinetic results of the reactions such as OH + H_2_O_2_ from IUPAC recommendation,^18^ and the indirect determinations of concentration for the reaction species might cause additional and unknown systematic errors. Direct quantification of both precursors and free radicals in the reaction cell would thus be crucial to perform accurate reaction kinetic studies, particularly for the radical–radical reactions. Recently, we have carried out the measurements on absolute line strength of the fundamental transitions of H_2_O_2_ and OH as well as the rate coefficient of the reaction OH + H_2_O_2_ using synchronized two-color time-resolved dual-comb spectroscopy in the mid-infrared spectral ranges.^19^ Utilizing two sets of dual-comb spectrometers near 7.9 and 2.9 μm, high-resolution time-resolved spectra of H_2_O_2_ and OH were simultaneously measured before and after 248 nm photolysis of the flowing mixtures of H_2_O_2_/N_2_ at varied conditions to investigate the kinetics of the reaction between OH and H_2_O_2_. By analyzing the temporal profiles of OH with the kinetic model including self-reactions of the OH and HO_2_ and taking into account the reaction of OH + HO_2_ with the rate coefficients of 1 × 10^–11^ and 1 × 10^–10^ cm^3^ molecule^–1^ s^–1^, the rate coefficients for the reaction OH + H_2_O_2_ were respectively determined to be (1.97 ± 0.10) × 10^–12^ and (1.92 ± 0.10) × 10^–12^ cm^3^ molecule^–1^ s^–1^ at 296 K, which are in excellent agreement with the recent experimental value of (2.00 ± 0.15) × 10^–12^ cm^3^ molecule^–1^ s^–1^ at 298 K, reported by Jiménez et al.,^20^ but up to 16% higher than the value of 1.7 × 10^–12^ cm^3^ molecule^–1^ s^–1^ from the IUPAC recommendation.^18^

In this work, to further explore the kinetics of the reaction between OH and HO_2_, hydrogen peroxide (H_2_O_2_) was chosen as the precursor for generation of OH radicals upon 248 nm laser photolysis, because the photodissociation of H_2_O_2_ has been well evaluated in previous experiments.^21−23^ The quantum yield for OH production was determined to be two in the 248 nm photolysis system,^21,22^ and no evidence for vibrationally excited OH was observed on laser irradiation of H_2_O_2_ at 222 nm.^23^ By employing photodissociation of H_2_O_2_ at 248 nm (R1), the OH radicals can be generated immediately and they will subsequently react with H_2_O_2_ to form H_2_O and HO_2_ radicals (R2). In the meantime, radical–radical reactions such as OH + HO_2_ (R3), OH + OH (R4), and HO_2_ + HO_2_ (R5) will occur as well. The rate coefficients of these involved reactions are shown in Table 1 and will be used in the kinetic model simulation. Because the rate coefficients for the self-reactions of OH and HO_2_ (R4 and R5) are much slower compared to that of the reaction OH + HO_2_ (R3) by 1–2 orders of magnitude, the reaction OH + HO_2_ (R3) can be considered as the dominant secondary reaction in the OH + H_2_O_2_ reaction system and plays a major role influencing the yield of HO_2_ radicals. Based on the clear kinetic model, we were able to investigate the kinetics of the reaction OH + HO_2_ through direct determination of precursor (H_2_O_2_) and radical (OH and HO_2_) concentrations in a flash photolysis reactor and simultaneous analysis of measured temporal profiles of the OH and HO_2_ radicals.

**Table 1:** Reactions and Rate Coefficients Employed in the Kinetic Model Simulation

	reaction	rate coefficient^a^	ref.
R1	H_2_O_2_ + *hν*_248_→ 2OH		(21−23)
R2	OH + H_2_O_2_ → H_2_O + HO_2_	1.92 × 10^–12^	(19)
R3	OH + HO_2_ → H_2_O + O_2_	*k*_OH+HO_2__, determined in this work	
R4a	OH + OH → H_2_O + O	1.48 × 10^–12^	(18)
R4b	OH + OH + M → H_2_O_2_ + M	6.9 × 10^–31^ × [N_2_]	(18)
R5	HO_2_ + HO_2_ + M → H_2_O_2_ + O_2_ + M	1.6 × 10^–12^ + 5.2 × 10^–32^ × [N_2_]	(18)

a Rate coefficient in cm^3^ molecule^–1^ s^–1^; [N_2_] in molecules cm^–3^.

The schematic of the experimental setup is shown in Figure S2. In the experiment, we determined well the water-free rate coefficient for the OH + HO_2_ reaction and further explored the reaction kinetics under the conditions in the presence of water. The water-free gaseous H_2_O_2_ was prepared by utilizing thermal decomposition of the H_2_O_2_–urea complex which has been adopted in various experiments for generation of stable and gaseous H_2_O_2_.^24,25^ By warming up a glass container containing a mixture of H_2_O_2_–urea powder and SiO_2_ in the water bath to ∼45 °C, water-free gaseous H_2_O_2_ can be produced stably, and then it was carried by a stream of nitrogen (N_2_) and probed through a UV absorption cell before being introduced to the main reactor. The mixing ratio of the H_2_O_2_/N_2_ mixtures in the UV absorption cell was determined based on the measured absorption spectra and the absorption cross section of H_2_O_2_ in 215–220 nm region.^26^ Other streams of N_2_ were used for purging the windows and mirrors of the multipass cell and to dilute the concentration of H_2_O_2_ in the reactor. The initial concentration of H_2_O_2_, [H_2_O_2_]_0_, in the reactor was estimated by using the flow rate of each stream, mixing ratios of the H_2_O_2_/N_2_ premixtures, and the total pressure of the reactor. Furthermore, the [H_2_O_2_]_0_ was also directly determined by using the high-resolution absorption spectra and the line strengths of several H_2_O_2_ ν_6_ transitions^19^ for double confirming that no loss of H_2_O_2_ was caused in the flowing system. By monitoring the precursor mixtures with both UV and IR spectroscopy, we confirmed that the pure H_2_O_2_ can be produced without other impurities such as H_2_O, HNO_3_, and HNCO^27^ from the H_2_O_2_–urea/SiO_2_ mixture after 30 min of warming up and during stable heating at below 50 °C.

To directly determine the concentrations of H_2_O_2_ and H_2_O (for the experiments in the presence of water) inside the reactor before starting the time-resolved measurements and to simultaneously record the time-dependent absorption spectra of OH and HO_2_ radicals during reaction processes, two sets of dual-comb sources in the short-wave (near 2.9 μm) and long-wave (near 7.9 or 8.9 μm) mid-infrared spectral regions were operated and coupled into the multipass cell. The flash photolysis reactor was designed with a multipass path length of ∼41.8 m for the dual-comb beams and an overlap path of ∼13.4 m between the photolysis and dual-comb beams.^19,28^

Employing dual-comb spectrometers near 2.9 and 8.9 μm, high-resolution time-resolved spectra of OH and HO_2_ radicals were simultaneously measured upon 248 nm irradiation of the flowing mixture of H_2_O_2_/N_2_ ([H_2_O_2_]_0_ = 1.91 × 10^15^ molecules cm^–3^, *P*_T_ = 30.3 Torr, 296 K), as shown in Figure 1. Both OH and HO_2_ can be probed with the detection limit down to ∼1 × 10^11^ molecules cm^–3^ at the time resolution of tens of μs. In high-resolution time-resolved dual-comb spectroscopy, the spectral sampling spacing is equal to the repetition frequency of the dual-comb source, and the spectral sampling points can be increased by interleaving several dual-comb spectra measured with different spectral sampling spacings or varied central wavelengths. The temporal resolution of time-resolved dual-comb spectra is adjustable from the μs to ms level, depending on the length of measured interferogram used for generation of each time-dependent Fourier transform dual-comb spectrum. A detailed description of the experimental approach with time-resolved dual-comb spectroscopy has been presented in our previous works.^28−30^

**Figure 1:**
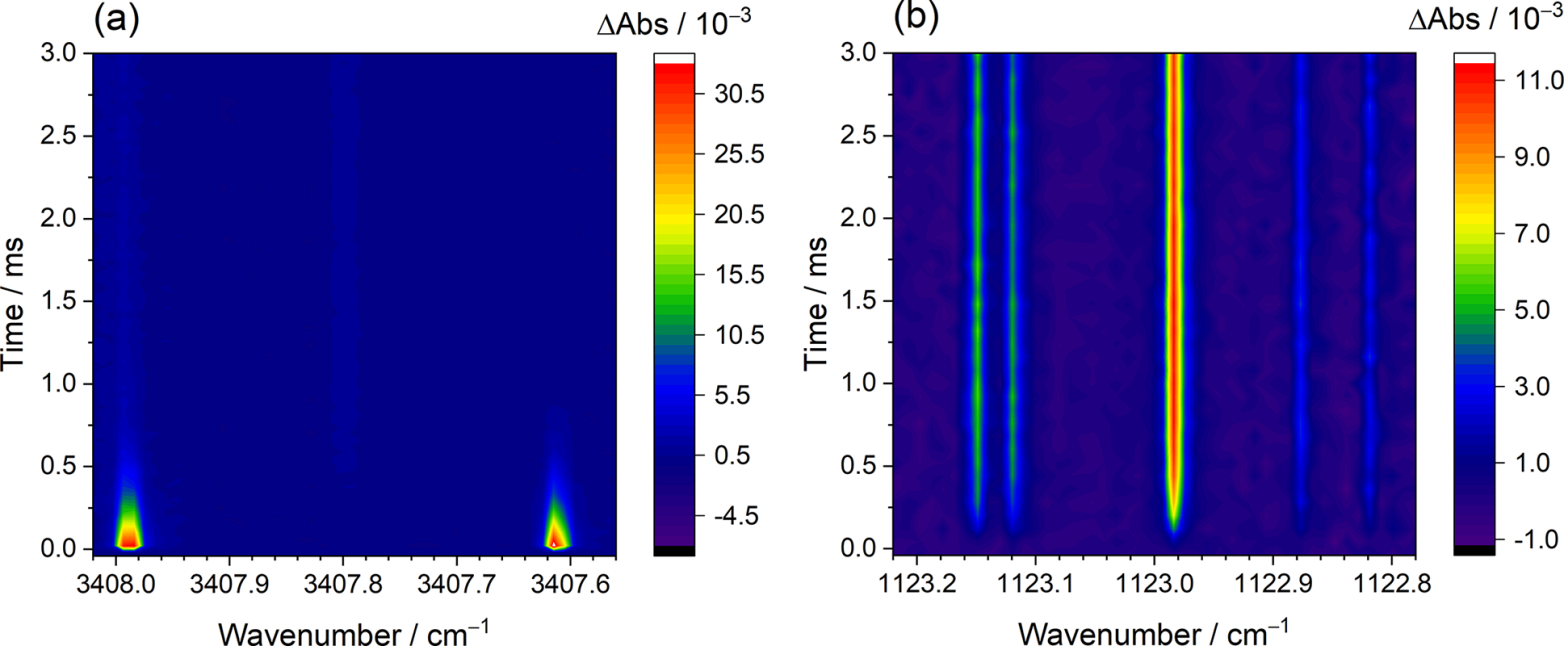
Time-resolved dual-comb spectra in the regions (a) 3407.56–3408.02 cm^–1^ and (b) 1122.78–1123.22 cm^–1^. The spectra were recorded simultaneously upon photolysis of a flowing mixture of H_2_O_2_/N_2_ ([H_2_O_2_]_0_ = 1.91 × 10^15^ molecules cm^–3^, *P*_T_ = 30.3 Torr, 296 K) over 10000 excimer laser shots at 248 nm with the photolysis energy of 38 mJ cm^–2^. Here, the spectral sampling spacing is 291 MHz (9.7 × 10^–3^ cm^–1^). The temporal resolutions of panels a and b are set to be 40 and 80 μs, respectively.

Figure 2 shows the comparison of the dual-comb spectra obtained before and after 248 nm irradiation of the flowing mixture of H_2_O_2_/N_2_ ([H_2_O_2_]_0_ = 1.91 × 10^15^ molecules cm^–3^, *P*_T_ = 30.3 Torr, 296 K). Figures 2a and 2d exhibit the absorbance spectra of H_2_O_2_ obtained before laser photolysis in the regions of 3407.52–3408.08 cm^–1^ (near 2.9 μm) and 1122.78–1123.22 cm^–1^ (near 8.9 μm), respectively. Although some absorption lines of H_2_O_2_ were observed near 2.9 μm, their line strengths are much smaller compared to that of OH by 2–3 orders of magnitude, and thus, the difference absorbance spectra would not be interfered by H_2_O_2_. After photolysis of the precursor mixture, transient infrared absorption spectra of two OH lines were identified at 3407.612 and 3407.989 cm^–1^ in the early reaction time (Figure 2b) and several HO_2_ absorption lines were observed in both spectral regions at the later reaction time, as shown in Figure 2c,e,f. To avoid the interference from the absorption of HO_2_ near 3408 cm^–1^, we mainly analyzed the temporal profiles of OH by monitoring the absorption line of OH at 3407.612 cm^–1^ with the measured line strength of (3.38 ± 0.25) × 10^–20^ cm molecule^–1^.^19^ The temporal profiles of HO_2_ can be extracted from the time-resolved spectra of the five absorption peaks of HO_2_ in the region of 1122.78–1123.22 cm^–1^. The total line strength of these five peaks were currently determined to be (4.18 ± 0.17) × 10^–20^ cm molecule^–1^ by simultaneous analysis of high-resolution time-resolved spectra of HCl and HO_2_ in the Cl + CH_3_OH reaction system. For precise determination of the concentration temporal profiles of OH and HO_2_ radicals, multiple measurements of time-resolved dual-comb spectra with different spectral sampling spacings were conducted to increase the spectral sampling points of the rotationally resolved infrared absorption spectra of OH and HO_2_. Figures S3 and S4 show the interleaved dual-comb spectra of OH and HO_2_, respectively. These spectra were curve-fitted with the multipeak Voigt function to obtain the integrated absorption area of each absorption transition, which can be further used to derive the time-dependent concentrations of OH and HO_2_. Based on the well-determined line strengths of OH and HO_2_ and their effective absorption path, the time-dependent concentration of each radical can be accurately determined with an uncertainty less than 10%. Moreover, the overall systematic errors could be reduced through simultaneous measurements of OH and HO_2_ and using the same effective absorption path.

**Figure 2:**
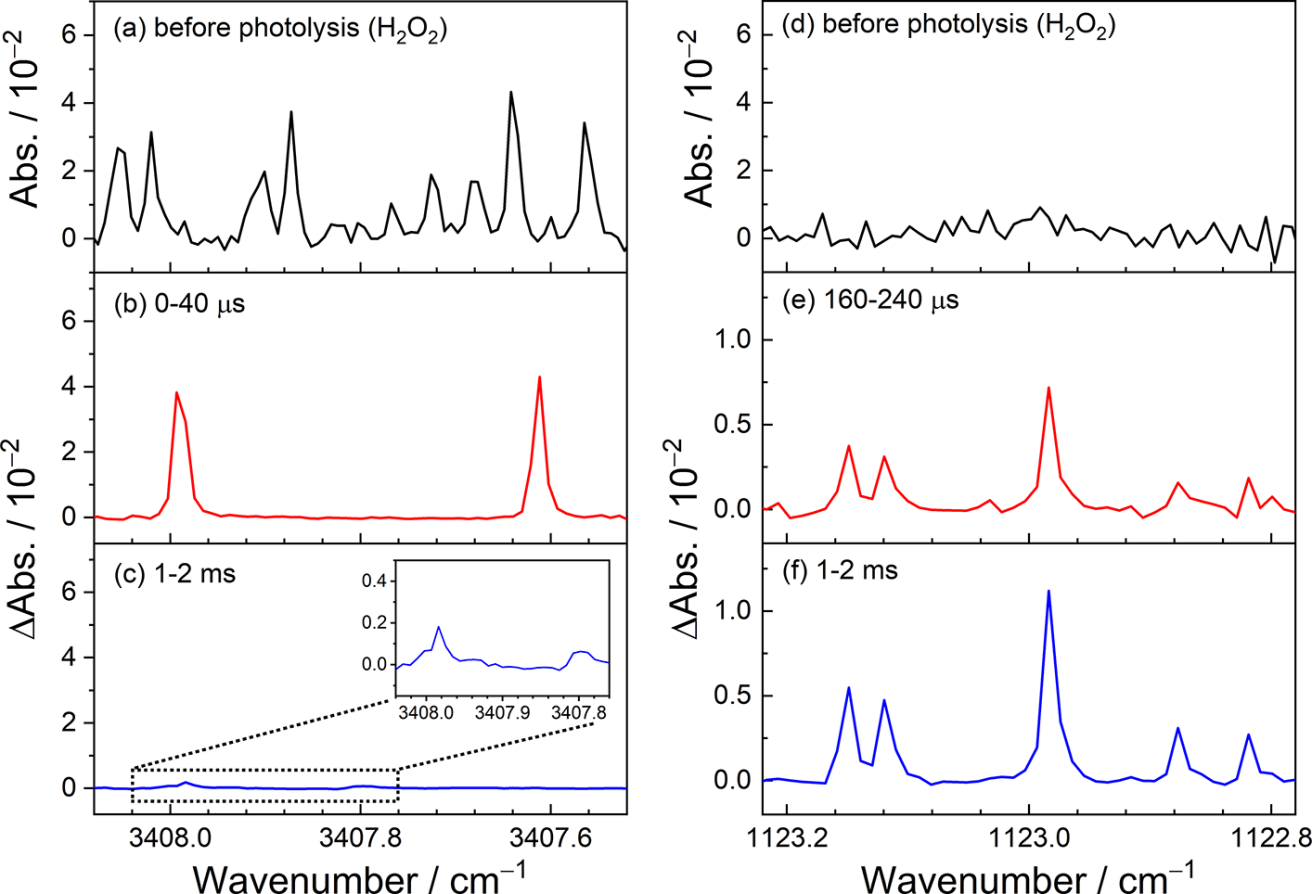
Comparison of the dual-comb spectra obtained before and after photolysis of a flowing mixture of H_2_O_2_/N_2_ ([H_2_O_2_]_0_ = 1.91 × 10^15^ molecules cm^–3^, *P*_T_ = 30.3 Torr, 296 K) in the regions (a–c) 3407.52–3408.08 cm^–1^ and (d–f) 1122.78–1123.22 cm^–1^. The spectra in panels a and d were recorded before the 248 nm irradiation. The difference absorbance spectra were extracted at 0–40 μs (b), 160–240 μs (e), and 1–2 ms (c and f) from the measured time-resolved dual-comb spectra (Figure 1). The insert in panel c depicts the enlarged spectra in region 3407.76–3408.04 cm^–1^.

To further evaluate the kinetics of the radical–radical reaction between OH and HO_2_, over 24 measurements in six sets were conducted under the water-free conditions with the initial concentrations of hydrogen peroxide [H_2_O_2_]_0_ ≈ 9.7 × 10^14^ and 1.9 × 10^15^ molecules cm^–3^; photolysis energies *E*_248 nm_ = 19.0, 28.5, and 38.0 mJ cm^–2^; and the total pressure *P*_T_ ≈ 30.3 Torr at 296 K. A summary of experimental conditions of each experimental set is listed in Table S1. Figure 3 shows the comparison of the measured and simulated time traces of OH and HO_2_ under various conditions. The concentration temporal profiles of OH were obtained by analyzing the time-resolved spectra of the OH X^2^Π_3/2_ (1 ← 0) P(4.5)f transition at 3407.612 cm^–1^, and the HO_2_ time traces were obtained by analyzing the time-resolved spectra of five absorption peaks near 1123 cm^–1^. All concentration profiles were simultaneously analyzed using the kinetic model, taking into account the reactions of OH + H_2_O_2_, OH + HO_2_, and the self-reactions of OH and HO_2_, as listed in Table 1. For these experiments carried out at 30.3 Torr and 296 K, the diffusion rates of OH and HO_2_ were estimated below 10 s^–1^ and could be neglected in the kinetic analysis. In order to fit all concentration time traces of OH and HO_2_ obtained at varied conditions with the kinetic simulations, the rate coefficient of the reaction OH + HO_2_ was evaluated to be 1.10 × 10^–10^ cm^3^ molecule^–1^ s^–1^ at 296 K. Moreover, an additional two sets of experiments were carried out by determining the OH concentrations using another transition near 3484 cm^–1^ and measurement of HO_2_ concentrations with the spectra near 1123 cm^–1^ to double confirm the proposed results, as shown in Figure S5. According to the kinetic simulations, the 5% error of the measured *k*_OH+H_2_O_2__ could cause the uncertainty of 5% on the determination of *k*_OH+HO_2__. The influences on *k*_OH+HO_2__ caused by the possible errors of the self-reactions of OH and HO_2_ (30%) were also considered and estimated to be less than 1%. In addition, by simultaneous analysis of the OH and HO_2_ time traces obtained under different experimental conditions, the uncertainty of *k*_OH+HO_2__ caused by the errors of the line strengths of OH (7%) and HO_2_ (4%), spectral analysis (4%) as well as the effective absorption path (7%) was evaluated to be ∼9%. Considering all possible errors, the overall uncertainty of *k*_OH+HO_2__ was estimated to be ∼11% and the rate coefficient *k*_OH+HO_2__ was hence obtained to be (1.10 ± 0.12) × 10^–10^ cm^3^ molecule^–1^ s^–1^ at 296 K, which is in excellent agreement with the result reported by Assaf et al.,^6^ but it is ∼10 times larger than the most recent experimental value from Speak et al.^7^

**Figure 3:**
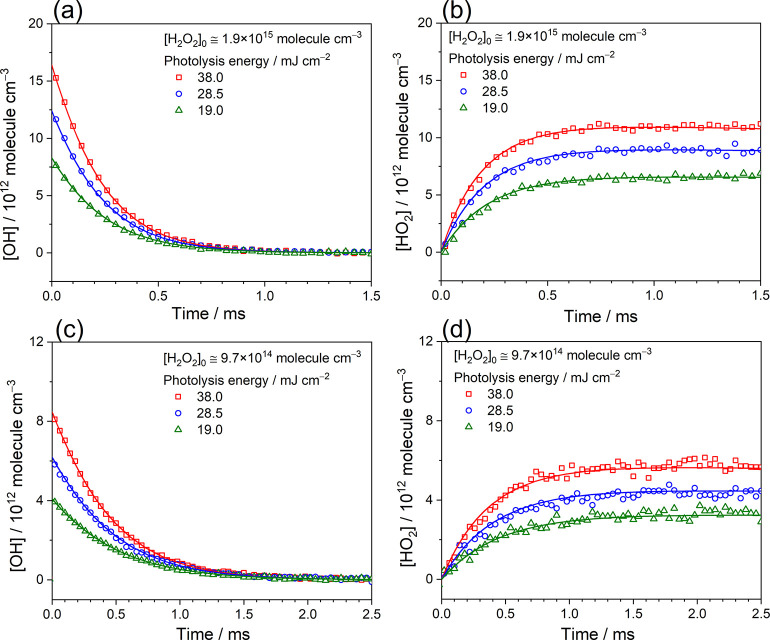
Comparison of the measured and the simulated temporal profiles of OH and HO_2_ at varied conditions. The concentration temporal profiles of (a) OH and (b) HO_2_ were recorded after the 248 nm irradiation of the flowing mixture of H_2_O_2_/N_2_ ([H_2_O_2_]_0_ ≈ 1.9 × 10^15^ molecules cm^–3^, *P*_T_ = 30.3 Torr, 296 K) with different photolysis energies. The concentration temporal profiles of (c) OH and (d) HO_2_ were recorded after the 248 nm irradiation of the flowing mixture of H_2_O_2_/N_2_ ([H_2_O_2_]_0_ ≈ 9.7 × 10^14^ molecules cm^–3^, *P*_T_ = 30.3 Torr, 296 K) with different photolysis energies. Here, the OH time traces were obtained by analyzing the time-resolved spectra of the OH X^2^Π_3/2_ (1 ← 0) P(4.5)f transition at 3407.612 cm^–1^ and the HO_2_ time traces were obtained by analyzing the time-resolved spectra of five absorption peaks near 1123 cm^–1^. The open symbols represent the measured temporal profiles with a resolution of 40 μs. The solid lines represent the simulated profiles using the kinetic model, as shown in Table 1, with *k*_OH+HO_2__ = 1.10 × 10^–10^.

Moreover, experiments in the presence of water were also conducted in this work to explore the possible enhancement effect on the rate coefficient of the reaction OH + HO_2_. By employing rotationally resolved infrared absorption spectra, the concentrations of both H_2_O_2_ and H_2_O in the reactor were calibrated and measured directly. Figure 4 shows the concentration profiles of OH and HO_2_ which were measured after 248 nm irradiation of the flowing mixture of H_2_O_2_/H_2_O/N_2_ ([H_2_O_2_]_0_ = 1.41 × 10^15^ molecules cm^–3^, [H_2_O]_0_ = 6.32 × 10^16^ molecules cm^–3^, *P*_T_ = 31.6 Torr, 296 K) with the photolysis energy of 33.3 mJ cm^–2^. The comparison of measured time traces and simulated profiles derived using the kinetic model with different *k*_OH+HO_2__ values is also displayed in Figure 4. The long-dashed, solid, and short-dashed curves represent the simulated profiles derived using the kinetic model, as shown in Table 1, with *k*_OH+HO_2__ = 0.2 × 10^–10^, 1.1 × 10^–10^, and 2.0 × 10^–10^ cm^3^ molecule^–1^ s^–1^, respectively. Because the yields of HO_2_ are strongly sensitive to the value of *k*_OH+HO_2__ in the kinetic simulations, *k*_OH+HO_2__ can be clearly determined. The measured OH and HO_2_ time traces were both fitted well with the kinetic simulations using the rate coefficient *k*_OH+HO_2__ of 1.10 × 10^–10^ cm^3^ molecule^–1^ s^–1^, indicating that the rate coefficient of the reaction OH + HO_2_ would not be enhanced at the conditions in the presence of water.

**Figure 4:**
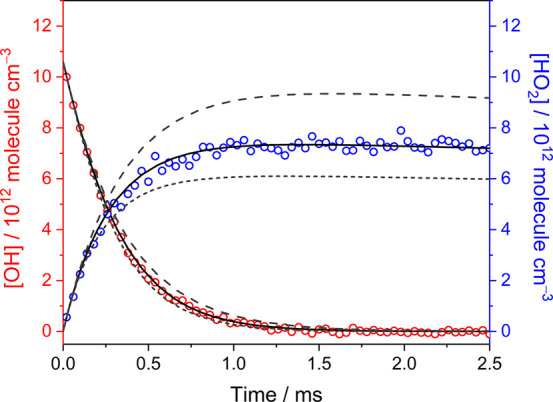
Temporal concentration profiles of OH (red open symbol) and HO_2_ (blue open symbol) obtained at the condition in the presence of H_2_O. The concentration profiles were measured after 248 nm irradiation of the flowing mixture of H_2_O_2_/H_2_O/N_2_ ([H_2_O_2_]_0_ = 1.41 × 10^15^ molecules cm^–3^, [H_2_O]_0_ = 6.32 × 10^16^ molecules cm^–3^, *P*_T_ = 31.6 Torr, 296 K) with the photolysis energy of 33.3 mJ cm^–2^. The solid curves represent the simulated profiles derived using the kinetic model, as shown in Table 1, with *k*_OH+HO_2__ = 1.1 × 10^–10^ cm^3^ molecule^–1^ s^–1^. The long-dashed and short-dashed lines represent the simulated profiles derived using the kinetic model, as shown in Table 1, with *k*_OH+HO_2__ = 0.2 × 10^–10^ and 2.0 × 10^–10^ cm^3^ molecule^–1^ s^–1^, respectively.

Figure 5 displays the comparison of the rate coefficient *k*_OH+HO_2__ obtained from different experiments at near room temperature,^6−14^ and the detailed descriptions of these experiments are summarized in Table S2. The rate coefficients *k*_OH+HO_2__ are displayed in the range of (0.1–1.2) × 10^–10^ cm^3^ molecule^–1^ s^–1^, and the maximum discrepancy of 13.5σ for the *k*_OH+HO_2__ is observed between the two more recent experiments which were respectively conducted by Assaf et al.^6^ and Speak et al.^7^ Owing to the high reactivity of OH radicals and indirect measurements of HO_2_ radicals, many studies expended considerable effort on calibration and estimation of radical concentrations through various methods and employed multiple reactions to perform cross comparisons. Each experiment would have its systematic errors, and the additional uncertainty could be increased through the indirect determination based on kinetics of multiple reactions, because each reaction might be carried out using different systems in different groups.

**Figure 5:**
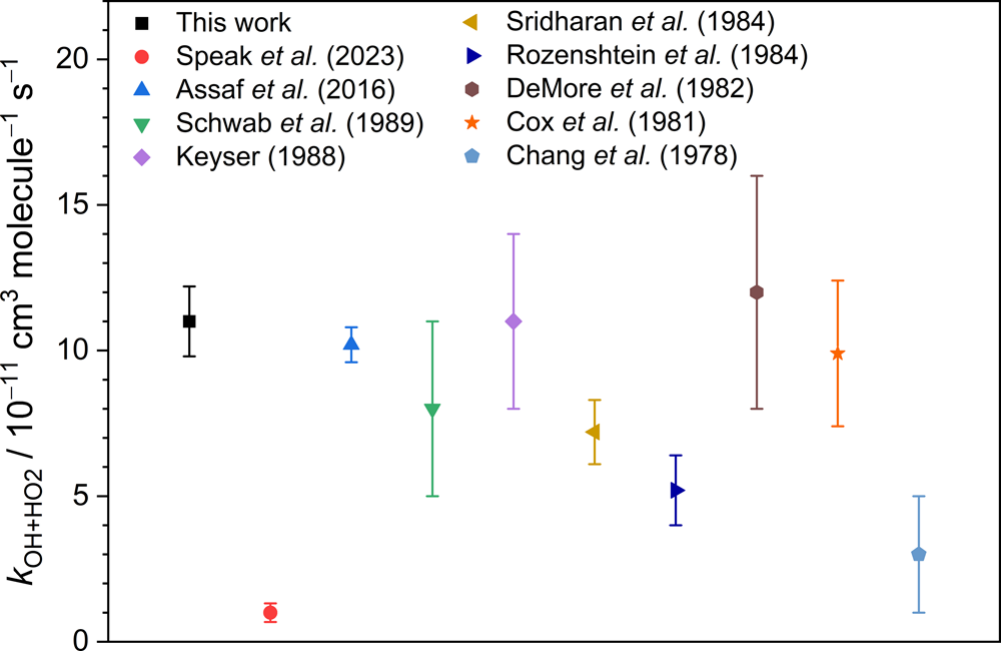
Comparison of the experimental results of rate coefficient *k*_OH+HO_2__, at 295–308 K. A summary of methods, conditions, and results of each experiment is listed in Table S2.

In conclusion, we have accomplished the accurate measurement of the kinetics of the OH + HO_2_ radical–radical reaction through direct determination of precursor and radical concentrations in the reaction system of the 248 nm photolysis of hydrogen peroxide. Employing synchronized two-color dual-comb spectroscopy, multiple species, including H_2_O_2_, H_2_O, OH, and HO_2_, can be directly quantified based on the measured high-resolution infrared absorption spectra. Most importantly, all key parameters such as the rate coefficient of the reaction OH + H_2_O_2_ and absolute line strengths of H_2_O_2_, OH, and HO_2_ were also measured using the same experimental system to reduce the systematic errors and to avoid unknown uncertainty. The water-free rate coefficient for the OH + HO_2_ reaction was determined to be (1.10 ± 0.12) × 10^–10^ cm^3^ molecule^–1^ s^–1^ at 296 K, and no enhancement in the rate coefficient was observed in the experiments with the addition of water. This work as an independent experiment that was implemented based on direct quantitation of all key reactants and careful kinetic evaluations is important and essential for reassessing the rate coefficient of the reaction between OH and HO_2_. Moreover, the proposed approach, which is capable of achieving direct measurement of precursor, radical, and product concentrations in the reactor, opens up opportunities for accurate kinetic studies of the complex radical–radical reactions.

## Supporting Information Available

Description of previous investigations of the OH + HO_2_ reaction, schematic of the experimental setup, difference absorbance spectra of OH and HO_2_ with fitted Voigt curves, comparison of the measured and the simulated temporal profiles of OH and HO_2_, a summary of experimental conditions of each experimental set, and a summary of different experiments determining the rate coefficient of the OH + HO_2_ reaction (PDF)
Transparent Peer Review report available (PDF)
