# Supplementary material for: Accurate Kinetic Studies of OH + HO2 Radical–Radical Reaction through Direct Measurement of Precursor and Radical Concentrations with High-Resolution Time-Resolved Dual-Comb Spectroscopy
Source: J Phys Chem Lett. 2024 Mar 28;15(14):3733–9. doi: 10.1021/acs.jpclett.4c00494 (PMC11017308; doi:10.1021/acs.jpclett.4c00494)
Supplement: Supplementary file 1 — jz4c00494_si_001.pdf [file jz4c00494_si_001.pdf]

Supporting Information

Accurate Kinetic Studies of OH + HO_2 Radical–Radical Reaction through Direct Measurement of Precursor and Radical Concentrations with High-Resolution Time-Resolved Dual-Comb Spectroscopy

I-Yun Chen[1,2], Che-Wei Chang[1,3,4], Christa Fittschen[5], Pei-Ling Luo*[,1]

[1] Institute of Atomic and Molecular Sciences Academia Sinica, Taipei 106319, Taiwan.
[2] Department of Chemistry, National Taiwan University, Taipei, 10617, Taiwan.
[3] Molecular Science and Technology Program, Taiwan International Graduate Program, Academia Sinica, 11529, Taipei, Taiwan.
[4] International Graduate Program of Molecular Science and Technology, National Taiwan University, 10617, Taipei, Taiwan.
[5] University Lille, CNRS, UMR 8522, PC2A–Physicochimie des Processus de Combustion et de l'Atmosphère, F-59000 Lille, France.

*corresponding author: plluo@gate.sinica.edu.tw

Table of content

Note S1. Description of previous investigations of the OH + HO_2 reaction.
Figure S1. Comparison of the experimental and theoretical results of the rate coefficient k_{OH+HO2}, at around 300 K.
Figure S2. Schematic of the experimental setup.
Figure S3. Difference absorbance spectra of OH with fitted Voigt curve.
Figure S4. Difference absorbance spectra of HO_2 with fitted Voigt curve.
Figure S5. Comparison of the measured and the simulated temporal profiles of OH and HO_2 at varied conditions. Here, the OH was measured near 3484 cm^{-1} and HO_2 was measured near 1123 cm^{-1}.
Table S1. Summary of experimental conditions of each experimental set.
Table S2. Summary of experimental conditions, methods and results for determination of the rate coefficient of the reaction OH + HO_2.
References

Note S1. Description of previous investigations of the OH + HO_2 reaction.

The radical-radical reaction rate coefficient between OH and HO_2 has been investigated by both experimental and theoretical studies.[1–16] Figure S1 shows the comparison of the rate coefficient k_{OH+HO2} obtained from previous experimental and theoretical studies at around 300 K.

In previous experiments, the reaction rate coefficients fall in the range of (0.1–1.2)×10^{-10} cm^3 $molecule^{-1}$ s^{-1} at around 300 K, as shown in Figure S1(a).[1–9] According to the most recent experiment by Speak *et al.*, a rather small rate coefficient, k_{OH+HO2} = (1.00±0.32)×10^{-11} cm^3 $molecule^{-1}$ s^{-1}, was obtained by measuring the temporal profile of OH and HO_2 upon 248-nm photolysis of H_2O_2 (50% v/v with water) in low pressure condition based on laser-induced fluorescence (LIF).[1] This value was smaller by an order of magnitude comparing to k_{OH+HO2} = (1.02±0.06)×10^{-10} cm^3 $molecule^{-1}$ s^{-1} reported by Assaf *et al.*,[2] which was determined based on time profiles of OH and HO_2 generated in the 248-nm photolysis of H_2O_2/$(COCl)_2$/CH_3OH/O_2 mixture by using CW cavity ring-down spectroscopy as well as the LIF signal of OH. Other experiments were carried out by employing the microwave discharge,[3–6,9] or flash photolysis cells,[7,8] coupled with LIF,[5,7] resonance fluorescence (RF),[3,4,9] electron paramagnetic resonance (EPR) spectroscopy,[6] or UV absorption spectroscopy.[8] The detailed descriptions of these experiments are summarized in Table S2. Currently, the preferred value from IUPAC is 1.1×10^{-10} cm^3 $molecule^{-1}$ s^{-1}, which is mainly based on the experimental results reported by Keyser with an uncertainty of approximately 30%.[4]

For the theoretical studies, the reaction rate coefficients are scattered in the range of (0.1–1.7)×10^{-10} cm^3 $molecule^{-1}$ s^{-1} at 300 K, as shown in Figure S1(b).[1, 10–16] For the previous theoretical studies before 2018, the reaction rate coefficients are reported in the relatively small range of (0.5–1.0)×10^{-10} cm^3 $molecule^{-1}$ s^{-1} at 300 K. However, the rate coefficients of this reaction reported from two recent theoretical studies show a large discrepancy by a factor up to 15. In 2020, Liu *et al.* studied the kinetics of OH + HO_2 using ring polymer molecular dynamics (RPMD) and quantum dynamics (QD) methods. They first calculated the rate coefficient by employing the QD method, and the value was reported to be 3.5×10^{-11} cm^3 $molecule^{-1}$ s^{-1}. Furthermore, based on the Bannett-Chandler factorization, they obtained the RPMD rate coefficient with a value of 1.66×10^{-10} cm^3 $molecule^{-1}$ s^{-1}, which is the largest value in the theoretical studies.[10] In comparison to the results from Liu *et al.*, recently, Speak *et al.* (2023) calculated the water free potential energy surface of OH + HO_2 at various levels to obtain the rate coefficients.[1] The rate coefficients derived based on the RCCSD(T)-F12b/CBS//RCCSD/aug-cc-pVTZ level (method A) and the UCCSD(T)/CBS//UCCSD/aug-ccpVTZ level (method B) were reported to be 1.1×10^{-11} and 4.7×10^{-11} cm^3 $molecule^{-1}$ s^{-1}, respectively.

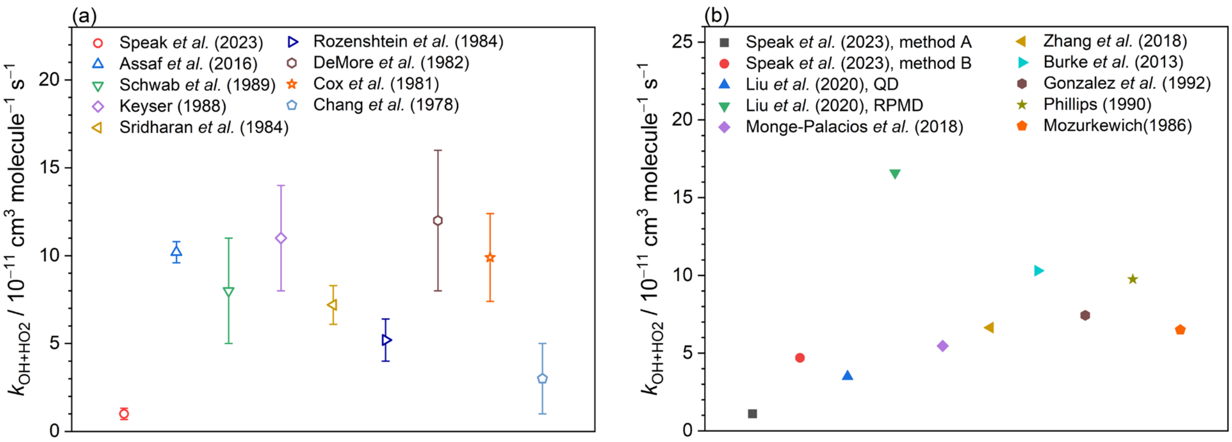

Figure S1. Comparison of the experimental and theoretical results of the rate coefficient k_{OH+HO2}, at around 300 K. (a) Comparison of the experimental results of k_{OH+HO2} at 295–308 K. A summary of methods, conditions, and results of each experiment are listed in Table S2. (b) Comparison of the theoretical results of k_{OH+HO2} at 300 K.

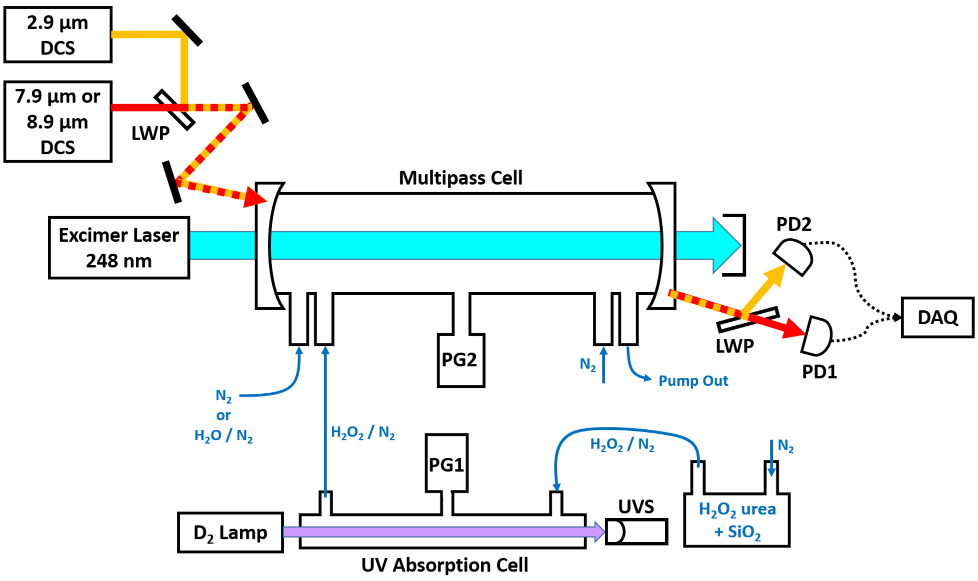

Figure S2. Schematic of the experimental setup. Here, DCS is the dual-comb source, LWP is the longwave pass filter, PG is the pressure gauge, PD is the photodiode, DAQ is the data acquisition board, and UVS is the ultraviolet spectrometer. To generate water-free H_2O_2, the urea hydrogen peroxide powder was mixed with an equivalent amount of dry sea sand (SiO_2) in a glass container to avoid agglomeration during the heating process in a water bath at ~45°C. Upon heating, a well-calibrated nitrogen gas flow would pass through the container at a flow rate of 1000 sccm to carry out the generated gaseous H_2O_2 into a 50-cm UV absorption cell before injection into the multipass cell. A D_2 lamp (StellarNet Inc., SL3) and a UV spectrometer (Oceanhood, XS11639) were used to obtain UV absorption spectra of H_2O_2 inside the 50-cm cell. The mixing ratio of the H_2O_2/N_2 mixtures inside the UV cell was evaluated by employing the measured UV absorption spectra and the absorption cross section of H_2O_2 in region 215–220 nm.[17–20] The concentration of H_2O_2, $[H_2O_2]_0$, inside the multipass cell can be estimated by using the flow rate of each stream, mixing ratios of the H_2O_2/N_2 pre-mixtures, and the total pressure of the reactor. The $[H_2O_2]_0$ were also directly measured by probing the H_2O_2 absorption lines near 1269 cm^{-1} before photolysis for double checking the initial concentration of H_2O_2 inside the reactor.

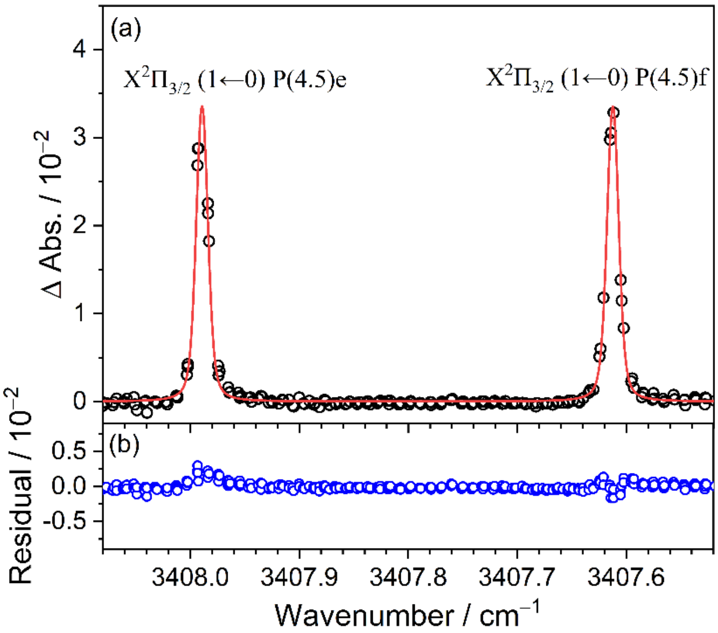

Figure S3. Difference absorbance spectra of OH with fitted Voigt curve. The spectrum (black open dots) was obtained by interleaving of four dual-comb spectra recorded with spectral sampling spacings of 279 MHz (0.93 × 10^{-3} cm^{-1}) or 291 MHz (0.97 × 10^{-3} cm^{-1}) at 0–40 μs after photolysis of the flowing mixture of H_2O_2/N_2 ($[H_2O_2]_0$ = 1.92×10^{15} molecule cm^{-3}, P_T = 30.3 Torr, 296 K) at 248 nm with the photolysis energy of 28.5 mJ cm^{-2}. The spectrum was curve-fitted with the multipeak Voigt function to derive the integrated absorption area of each absorption transition. The red line represents the fitted curve and the blue open dots represent the fitting residual. The line strength for one of $X^2\Pi_{3/2}$ (1 ← 0) P(4.5) doublet transitions was determined to be (3.38±0.25)×10^{-20} cm $molecule^{-1}$ in our previous work.[21]

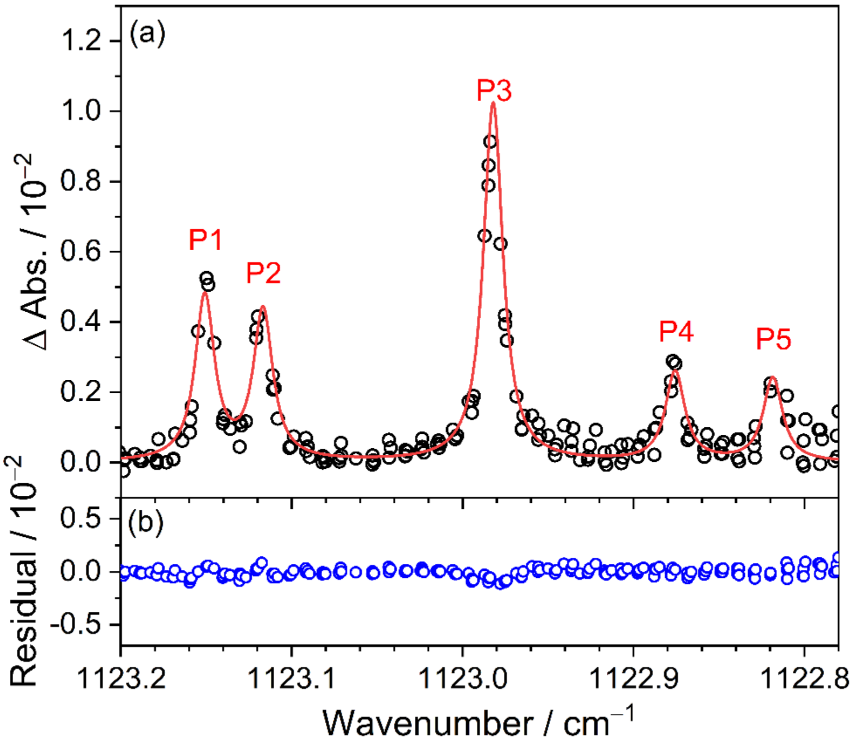

Figure S4. Difference absorbance spectra of HO_2 with fitted Voigt curve. The spectrum (black open dots) was obtained by interleaving of four dual-comb spectra recorded with spectral sampling spacings of 279 MHz (0.93×10^{-3} cm^{-1}) or 291 MHz (0.97×10^{-3} cm^{-1}) at 0.8–1.2 ms after photolysis of the flowing mixture of H_2O_2/N_2 ($[H_2O_2]_0 = 1.92\times10^{15}$ molecule cm^{-3}, P_T = 30.3 Torr, 296 K) at 248 nm with the photolysis energy of 28.5 mJ cm^{-2}. The spectrum was curve-fitted with the multipeak Voigt function to derive the integrated absorption area of each absorption peak. The red line represents the fitted curve and the blue open dots represent the fitting residual. Here, each peak contains two ν_3 fundamental transitions of HO_2: P1 includes $13_{3,11}\leftarrow12_{3,10}$ F_1 and $13_{3,10}\leftarrow12_{3,9}$ F_1; P2 includes $13_{3,11}\leftarrow12_{3,10}$ F_2 and $13_{3,10}\leftarrow12_{3,9}$ F_2; P3 includes $13_{1,13}\leftarrow12_{1,12}$ F_2 and $13_{1,13}\leftarrow12_{1,12}$ F_1; P4 includes $13_{4,10}\leftarrow12_{4,9}$ F_1 and $13_{4,9}\leftarrow12_{4,8}$ F_1; and P5 includes $13_{4,10}\leftarrow12_{4,9}$ F_2 and $13_{4,9}\leftarrow12_{4,8}$ F_2. The total line strength of the five peaks were currently determined to be $(4.18\pm0.17)\times10^{-20}$ cm molecule^{-1} by simultaneously measuring the high-resolution time-resolved spectra of HCl and HO_2 in the Cl + CH_3OH reaction system.

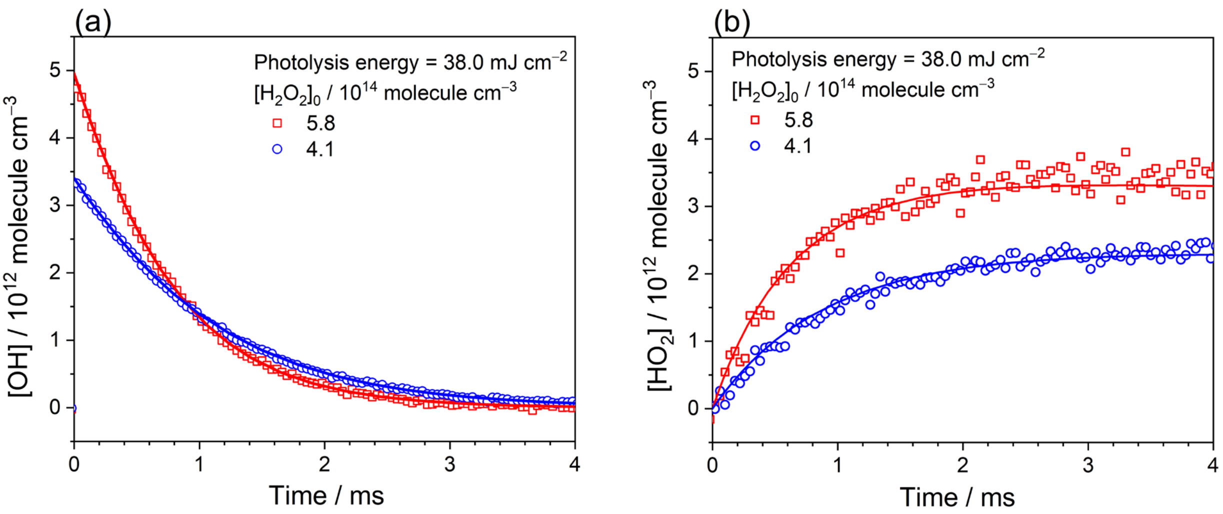

Figure S5. Comparison of the measured and the simulated temporal profiles of OH and HO_2 at varied conditions. The concentration temporal profiles of (a) OH and (b) HO_2 were recorded upon photolysis of the flowing mixtures of H_2O_2/N_2 ([H_2O_2]$_0$ = 5.8×10^{14} molecule cm^{-3}, P_T = 20.3 Torr, 296 K) (red) and H_2O_2/N_2 ([H_2O_2]$_0$ = 4.1×10^{14} molecule cm^{-3}, P_T = 15.3 Torr, 296 K) (blue) at 248 nm with the photolysis energy of 38.0 mJ cm^{-2}. The open symbols represent the measured temporal profiles with a time resolution of 40 μs. The solid lines represent the simulated profiles using the kinetic model, as shown in Table 1, with k_{OH+HO2} = 1.10×10^{-10}. Here, the OH time traces were obtained by analyzing the time-resolved spectra of the OH $X^2\Pi_{3/2}$ (1 ← 0) P(2.5) transitions near 3484 cm^{-1} and the HO_2 time traces were obtained by analyzing the time-resolved spectra of five absorption peaks near 1123 cm^{-1}.

Table S1. Summary of experimental conditions of each experimental set.

Set [a]	Temp. / K	P_T / Torr	Photolysis energy [e]	$[H_2O_2]_0$ / $10^{14\,f,g}$	$[H_2O]_0$ / $10^{16\,f,h}$	$[OH]_0$ / $10^{12\,f}$	$[HO_2]_{1.5\text{-}2.0\ ms}$ / $10^{12\,f}$
1 [b,d]	296	30.3	38.0	19.1	0	16.4	10.8
2 [b,d]	296	30.3	28.5	19.2	0	12.4	9.1
3 [b,d]	296	30.3	19.0	19.3	0	8.3	6.7
4 [b,d]	296	30.3	38.0	9.9	0	8.5	5.7
5 [b,d]	296	30.3	28.5	9.6	0	6.2	4.5
6 [b,d]	296	30.3	19.0	9.5	0	4.1	3.1
7 [b,d]	296	31.6	33.3	14.1	6.32	10.6	7.4
8 [c,d]	296	20.3	38.0	5.8	0	5.0	3.2
9 [c,d]	296	15.3	38.0	4.1	0	3.4	2.0

[a] Each set includes 4~5 individual experimental measurements.

[b] For the experiments, the OH $X^2\Pi_{3/2}$ (1←0) P(4.5) doublet transitions near 3407 cm^{-1} were probed.

[c] For the experiments, the OH $X^2\Pi_{3/2}$ (1←0) P(2.5) doublet transitions near 3484 cm^{-1} were probed.

[d] For the experiments, five absorption peaks of HO_2 near 1123 cm^{-1} were probed.

[e] in unit of mJ cm^{-2}.

[f] in unit of molecule cm^{-3}.

[g] The mixing ratio of the gaseous H_2O_2 in the bath gas N_2 before injection into the reactor was determined using UV absorption spectra and the absorption cross section of H_2O_2 in region 215–220 nm.[17–20] The $[H_2O_2]_0$ in the reactor was estimated by using the flow rate of each stream, mixing ratios of the H_2O_2/N_2 pre-mixtures, and the total pressure of the reactor. In addition, the H_2O_2 absorption lines near 1269 cm^{-1} were probed before photolysis for double checking the concentration of H_2O_2 inside the reactor.

[h] For the experiments, the H_2O absorption lines near 1121 and 3410 cm^{-1} were probed before photolysis.

Table S2. Summary of experimental conditions, methods and results for determination of the rate coefficient of the reaction OH + HO_2.

Study	T / K	Pressure / Torr	Methods	k_{OH+HO2} / 10^{-11} [a]
This work	296	15.3–31.6	248-nm photolysis of water-free H_2O_2 to generate OH and HO_2 radicals. Determination of the initial concentration of H_2O_2 by UV and IR absorption spectra. Simultaneous measurement of the OH and HO_2 time-dependent spectra using mid-infrared two-color time-resolved dual-comb spectroscopy (TR-DCS).	11.0 ± 1.2
Speak *et al.* [1] (2023)	298	75–200	248-nm photolysis of H_2O_2 (50% v/v with H_2O) to generate OH and HO_2 radicals. Measurement of the OH and HO_2 time profiles based on laser-induced fluorescence (LIF) signals of OH.	1.00 ± 0.32
Assaf *et al.* [2] (2016)	298	50	248-nm photolysis of the mixture of $H_2O_2/(COCl)_2/CH_3OH/O_2$ to generate OH and HO_2 radicals with different concentrations. Simultaneous measurement of the OH and HO_2 time profiles by OH LIF and cw-cavity ring-down spectroscopy (cw-CRDS), respectively.	10.2 ± 0.6
Schwab *et al.* [3] (1989)	298	2	Microwave discharge of CF_4 in He to generate F atoms which will react with H_2O/H_2O_2 to generate OH/HO_2 radicals. Measurement of the OH and HO_2 time profiles by laser magnetic resonance (LMR); O, H, and OH time profiles obtained by resonance fluorescence (RF).	8 ± 3
Keyser [4] (1988)	299	1	Microwave discharge of CF_4/F_2 in He to generate F atoms which will react with H_2O/H_2O_2 to generate OH/HO_2 radicals. Measurement of the OH time profiles and the concentrations of HO_2 by resonance fluorescence (RF).	11 ± 3
Sridharan *et al.* [5] (1984)	296	2.5	Microwave discharge of CF_4 in He to generate F atoms which will react with H_2O_2 to generate HO_2 radical. OH radicals generated from F reacting with H_2O, where F comes from F_2 + H. Measurement of the OH time profiles by LIF. The HO_2 is first converted to OH then observed by LIF.	7.2 ± 1.1
Rozenshtein, *et al.* [6] (1984)	298	8–10	Microwave discharge of H_2 in He to generate H atoms which will react with O_2 to generate HO_2 radicals. The generated HO_2 radicals can further react with H atom to form OH radicals. Measurement of the OH time profiles by electron paramagnetic resonance (EPR).	5.2 ± 1.2
Demore *et al.* [7] (1982)	298	730	184.9-nm photolysis of H_2O with excessive O_2 to generate OH and HO_2 radicals. Measurement of the OH time profiles by LIF.	12 ± 4

Cox *et al.* [8] (1981)	308	760	254-nm photolysis of the $O_3/H_2O/O_2$ mixture in He, Ar, or N_2 to generate OH and HO_2 radicals. Measurement of the OH and HO_2 time profiles by UV absorption spectroscopy.	9.9 ± 2.5
Chang *et al.* [9] (1978)	295	2.1–3.7	OH radicals are formed from the reaction between H and NO. HO_2 radicals can then be generated from OH + O_3. Measurement of the OH time profiles by resonance fluorescence (RF). The HO_2 is first converted to OH then observed by RF.	3 ± 2

[a] in unit of cm^3 $molecule^{-1}$ s^{-1}.

References

(1) Speak, T. H.; Blitz, M. A.; Medeiros, D. J.; Seakins, P. W. New Measurements and calculations on the kinetics of an old reaction: $OH + HO_2 \rightarrow H_2O + O_2$. *J. Am. Chem. Soc. Au* **2023**, *3*, 1684–1694.

(2) Assaf, E.; Fittschen C. Cross section of OH radical overtone transition near 7028 cm^{-1} and measurement of the rate constant of the reaction of OH with HO_2 radicals. *J. Phys. Chem. A* **2016**, *120*, 7051–7059.

(3) Schwab, J. J.; Brune, W. H.; Anderson, J. G. Kinetics and mechanism of the hydroxyl + hydroperoxo reaction. *J. Phys. Chem.* **1989**, *93*, 1030– 1035.

(4) Keyser, L. F. Kinetics of the reaction $OH + HO_2$ yields $H_2O + O_2$ from 254 to 382 K. *J. Phys. Chem.* **1988**, *92*, 1193–1200.

(5) Sridharan, U. C.; Qiu, L. X.; Kaufman, F. Rate constant of the hydroxyl + perhydroxyl (HO_2) reaction from 252 to 420 K. *J. Phys. Chem.* **1984**, *88*, 1281–1282.

(6) Rozenshtein, V. B.; Gershenzon, Y. M.; Il'in, S. D.; Kishkovitch, O. P. Reactions of HO_2 with NO, OH and HO_2 studied by EPR/LMR spectroscopy. *Chem. Phys. Lett.* **1984**, *112*, 473– 478.

(7) DeMore, W. B. Rate constant and possible pressure dependence of the reaction $OH + HO_2$. *J. Phys. Chem.* **1982**, *86*, 121–126.

(8) Cox, R. A.; Burrows, J. P.; Wallington, T. J. Rate coefficient for the reaction $OH + HO_2 = H_2O + O_2$ at 1 atmosphere pressure and 308 K. *Chem. Phys. Lett.* **1981**, *84*, 217–221.

(9) Chang, J. S.; Kaufman, F. Upper bound and probable value of the rate constant of the reaction $OH + HO_2 \rightarrow H_2O + O_2$. *J. Phys. Chem.* **1978**, *82*, 1683–1687.

(10) Liu, Y.; Song, H.; Li, J. Kinetic study of the $OH + HO_2 \rightarrow H_2O+O_2$ reaction using ring polymer molecular dynamics and quantum dynamics. *Phys. Chem. Chem. Phys.* **2020**, *22*, 23657–23664.

(11) Monge-Palacios, M.; Sarathy S. M. *Ab initio* and transition state theory study of the OH + $HO_2 \rightarrow H_2O + O_2(^3\sum_g^-)$/ $O_2(^1\Delta_g)$ reactions: yield and role of $O_2(^1\Delta_g)$ in H_2O_2 decomposition and in combustion of H_2. *Phys. Chem. Chem. Phys.*, **2018**, *20*, 4478–4488.

(12) Zhang, T.; Lan, X.; Qiao, Z.; Wang, R.; Yu, X.; Xu, Q.; Wang, Z.; Jin, L.; Wang, Z. Role of the $(H_2O)_n$ (n= 1–3) cluster in the$HO_2 + HO \rightarrow {}^3O_2$+ H2O reaction: mechanisticand kinetic studies. *Phys. Chem. Chem. Phys.*, **2018**, *20*, 8152–8165.

(13) Burke, M. P.; Klippenstein S. J.; Harding, L. B. A quantitative explanation for the apparent anomalous temperature dependence of $OH + HO_2 = H_2O + O_2$ through multi-scale modeling. *Proc. Combust. Inst.*, **2013**, *34*, 547–555.

(14) Gonzalez, C.; Theisen, J.; Schlegel, H.B.; Hase, W. L.; Kaiser, E. W. Kinetics of the reaction between OH and HO_2 on the triplet potential energy surface *J. Phys. Chem.*, **1992**, *96*, 1767–1774.

(15) Phillips, L. F. Collision-theory calculations of rate constants for some atmospheric radical reactions over the temperature range 10-600 K. *J. Phys. Chem.*, **1990**, *94*, 7482–7487.

(16) M. Mozurkewich Reactions of HO_2 with free radicals. *J. Phys. Chem.*, **1986**, *90*, 2216–2221.

(17) Lin, C.-L.; Rohatgi, N. K.; DeMore, W. B. Ultraviolet absorption cross sections of hydrogen

peroxide. *Geophys. Res. Lett.* **1978,** *5*, 113–115.

(18) Molina, L. T.; Molina, M. J. UV absorption cross sections of HO_2NO_2 acid vapor. *J. Photochem.* **1981**, *15*, 97–108.

(19) Nicovich J. M.; Wine, P. H. Temperature-dependent absorption cross sections for hydrogen peroxide vapor. *J. Geophys. Res.* **1988**, *93*, 2417–2421.

(20) Vaghjiani G. L.; Ravishankara, A. R. Absorption cross sections of CH_3OOH, H_2O_2, and D_2O_2 vapors between 210 and 365 nm at 297 K. *J. Geophys. Res.* **1989**, *94(D3)*, 3487–3492.

(21) Chang, C-W.; Chen, I-Y.; Fittschen, C.; Luo P-L. Measurements of absolute line strength of the ν_1 fundamental transitions of OH radical and rate coefficient of the reaction OH + H_2O_2 with mid-infrared two-color time-resolved dual-comb spectroscopy. *J. Chem. Phys.* **2023**, *159*, 184203.
