# Supplementary material for: Accurate Kinetic Studies of OH + HO2 Radical–Radical Reaction through Direct Measurement of Precursor and Radical Concentrations with High-Resolution Time-Resolved Dual-Comb Spectroscopy
Source: J Phys Chem Lett. 2024 Mar 28;15(14):3733–9. doi: 10.1021/acs.jpclett.4c00494 (PMC11017308; doi:10.1021/acs.jpclett.4c00494)
Supplement: Supplementary file 2 — jz4c00494_si_002.pdf [file jz4c00494_si_002.pdf]

jz-2024-00494h.R1

Name: Peer Review Information for "Accurate Kinetic Studies of OH + HO₂ Radical–Radical Reaction through Direct Measurement of Precursor and Radical Concentrations with High-Resolution Time-Resolved Dual-Comb Spectroscopy"

First Round of Reviewer Comments

Reviewer: 1

Comments to the Author

Chen et al. employ a flash-photolysis multipass cell coupled with dual-comb spectroscopy in the short-wave (near 2.9 μm) and long-wave (near 7.9 or 8.9 μm) mid-IR regions to quantitatively measure the reaction species before and after laser photolysis. HOOH is produced via heating of urea-hydrogen peroxide. This is an excellent piece of work and suitable for JPC Letters.

Major advances reported: The authors measure k(T) for OH + HOO using direct detection of reactant precursor (HOOH) and radical species of title reaction (OH and HOO). The current paper reaffirms previous measurements from Assaf et al. (which involved one of the senior authors of this paper, CF). Through detailed kinetics analysis, the rate coefficient for OH + HOOH is also reported and is consistent with the literature (a discrepancy of 16% is noted, but this is a reasonable level of uncertainty). The authors also conclude there is no impact of water concentration on the rate coefficient for OH + HOO. A convincing plot of the current data compared with all measurements in the literature (Figure 5) is provided.

Immediate significance: This work reconciles a disparity in literature ascribed to issues in definition of initial conditions of reactant concentrations based mostly on the direct quantification of HOOH via rotationally resolved IR-absorption spectra. The rate coefficient for this reaction is important to combustion and atmospheric chemistry

Technical suggestions: Although outside the scope of work, a welcomed addition to this paper would be an examination of the influence of different colliders – ranging from He to carbon dioxide to assess the role, if any, that collision efficiency might play in the reported rate coefficients. Also, no comparison is made to theoretical treatment of this reaction, such as Burke et al., Proc. Combust. Inst., 2013. Why not? I realize this is an experimental-focused effort to resolve

experimental discrepancies, which it certainly accomplishes, but some comments on such a comparison, even if in the SM, would be useful.

Reviewer: 2

Comments to the Author

Review jz-2024-00494h

The work described in this manuscript is motivated by a clear discrepancy (a factor of 10) between measured values of the rate coefficient at room temperature for the reaction of two radicals, OH and HO_2. As both radicals play important roles in atmospheric chemistry and their cross reactions play a significant role in their removal from the upper troposphere, it is particularly important that the discrepancy be resolved.

The manuscript describes a beautiful collaboration between excellent investigators located nearly half a world apart. They apply a new experimental approach, time-resolved dual-comb spectroscopy, that for the first time directly measures the concentration of each radical spectroscopically. Moreover, the result clearly confirms the value of the rate coefficient reported previously by reference 6, contradicting the more recent report in reference 7. In addition, these experiments investigated the reported enhancement of the OH + HO_2 reaction by water as reported in reference 7, but the present work did not confirm the previous results.

My only significant criticism of the manuscript is that the description of the reaction mechanism is rather confusing and overly brief. Essentially the authors try to do that using only the first half of the last sentence on page 5 that continues onto page 6. I puzzled over this description until I found Table S1 in the supplemental material that contains the kinetic model and the rate coefficients. I recommend moving this table to the text and using it and two or three well-crafted sentences to clearly describe this most important information.

There are a couple of additional items that I would like to see included in the text.

1. It appears (see figure 3) that the 248 nanometer photolysis of H_2O_2 produces only 2OH and no H + HO_2 directly. Is that correct?

2. The concentration of the radicals were measured using their absorption spectrum. The particular lines, and the values of the absorption coefficients used, should be given in the text. This information would be helpful for anyone who wanted to repeat the experiment and would give all readers an idea of the relative accuracy of the concentration measurements of each radical.

Reviewer: 3

Comments to the Author

The authors present a study of the OH + HO2 reaction, and compare it with previous studies of the same reaction, in an attempt to consolidate findings and solidify a rate coefficient to be used for this reaction. The use of frequency comb spectroscopy is beneficial here, in that both HO2 and OH can be monitored. There are a few questions I think should be addressed in either the main manuscript or in the SI:

1. The use of the urea-H2O2 precursor: what role does the SiO2 play in the precursor preparation? Have there been previous studies on the thermal decomposition of the SiO2/urea-H2O2 mix that show H2O2 is the only gaseous compound evolved at this temperature? There should be a discussion or citation here to showcase that any thermal decomposition products (that could interfere with the OH + HO2 reaction) have been considered and discounted.

2. The concentration of OH relies on the measurements from the author's previous paper of line strengths. I have a few questions regarding this:

a) For the concentration of OH, the author's previous paper and this one relies on the assumption that the quantum yield of OH from 248 nm photolysis of H2O2 is 2. This is the recommended value, but the study from Nesbitt and coworkers (https://doi.org/10.1063/1.464735) does show a power dependence to the quantum yield, which shifts away from 2 as the power is increased. The authors have looked at multiple 248 nm laser powers -- do they see any power dependence to the quantum yield of OH?

b) The previous paper showed a consistent 25% disagreement of the line strength compared to that reported by HITRAN for the OH transition used here. If the authors used the line strengths reported by HITRAN instead of the ones derived in the previous paper, does that make a difference to the derived reaction rate coefficient?

3. It reads as though the authors used only one transition for OH to derive the final reaction rate coefficient (bottom of page 10). Is this correct? How sensitive is the measurement to which transition is chosen to be used? Do the authors use multiple transitions for HO2, or just a single one? It seems that the power of a frequency comb spectrometer lies in being able to use a

combined fit to multiple transitions to get a more accurate representation of the kinetics, so I'm curious if the authors took advantage of it.

4. If I recall correctly, OH is produced vibrationally cold but rotationally hot from 248 nm photolysis of H2O2. How is rotational relaxation accounted for in your kinetic analysis? Can you see evidence of any rotational relaxation based on multiple rovibrational transitions being probed? I believe rotational energy transfer rates for OH are on the order of 10^-10 cm3 molecule−1 s−1 (at least for helium, see https://iopscience.iop.org/article/10.1088/1361-6463/aa5b28/meta and references therein, such as reference 3), so perhaps the rotational relaxation is fast under your pressure conditions and you observe a thermalized distribution? Are you able to fit the rotational temperature of your observed OH?

5. As with question 1 above, some additional experimental detail would be appreciated, perhaps in the SI, or at least reference to the appropriate papers. For example, what is the residence time in the kinetic gas cell, and are there any issues around wall-loss (not included in the kinetic model)? Have the authors noticed a change in any of the measurements if they do not irradiate the H2O2 precursor in the UV absorption cell prior to its entrance into the gas cell used for kinetics (what is the D2 lamp make/model and its flux)?

Minor points:

1. Please remove the hyphen between the number and its unit for 248 nm.

2. "Summary" is misspelled in the caption for Figure 5.

Reviewer: 4

Comments to the Author

The paper by Chen et al is an experimental study, aimed at addressing a discrepancy in recent measurements of the OH + HO2 reaction rate coefficient. OH is produces by laser flash photolysis of H2O2/N2 mixtures in a flow cell; subsequent reaction of OH + H2O2 forms HO2, and the time-resolved yield of HO2 is sensitive to the reaction of OH + HO2. Both OH and HO2 are probed by multi-pass dual-frequency comb spectrometers in the mid-IR and far IR range. Quantification of OH and HO2 is possible via earlier vibrational line strength determinations by the same group. Recorded time traces at several H2O2 and initial radical concentrations are compared to kinetic model predictions, and from this the value for k(OH+HO2) is derived. The study is carefully conducted and clearly written. The major finding of the paper is that the rate coefficient k(OH+HO2) = (1.1+/-0.14)e-10 cm3/molecule s.

From a technical standpoint, this work represents state-of-the-art current capability of time-resolved frequency-comb probing of reactive radical species. However, the main scientific result is not a transformative new finding but rather a confirmation of the earlier value by one of the authors for a single rate coefficient of a single reaction (albeit an important one). Furthermore, the result agrees quantitatively with the current IUPAC recommendation. Therefore, although the present result increases our confidence in the rate coefficient for OH + HO2, it is not likely to impact modeling efforts.

Overall, this is a thorough and careful study that does not report an urgent new development and therefore would better fit into another journal like JPCA or IJCK. In addition, I would suggest several other points to assist the reader:

- On pages 5 – 6 the authors refer to a 2023 study by Chang et al (ref. 19) in which OH line absorption line strengths are quantified by referencing them to H2O2. The setup in that study seems very similar to this one, including the method of OH production and the probe method, dual-comb spectroscopy. What are the difference in the two studies, that make the previous study insensitive to OH + HO2 but the present one sensitive to this reaction?
- Please include a table of experimental conditions, including the initial radical concentrations (probably in the SI). Please consider reporting the reactant concentrations as number densities rather than partial pressures in Torr – it makes estimating reaction rates easier.
- Are the present results exclusively sensitive to OH + HO2, or could correlation with some other reactions significantly influence the determination of k(OH+HO2)? The authors should consider a sensitivity analysis using any available chemical modeling software like Chemkin or Cantera, in order to support the idea that the present results are mainly or exclusively sensitive to the reaction of interest.
- The experiment includes a UV spectrometer to measure [H2O2]. Do the IR dual-comb measurements of pre-photolysis [H2O2] agree with the UV measurements? Those are not mentioned in the paper.
- Is it possible to obtain time traces of H2O2 and H2O (under water-free conditions) from these measurements? Several reactions in the mechanism make H2O2 and H2O as products, and it would be interesting to compare those experimental results to model predictions, if it were possible.

Author's Response to Peer Review Comments:

We have responded all comments by the reviewers and made corresponding changes. Please find the revised manuscript, SI, and reply letter. I hope that the revised manuscript is acceptable for publication now.

Response and revisions to referees' comments

We appreciate very much the valuable comments by the reviewers. We have revised the manuscript accordingly and hope that this manuscript is now acceptable publication in The Journal of Physical Chemistry Letters. For convenience, we listed the response and revisions in blue font color after each comment.

Reviewer: 1

Comments:

Chen et al. employ a flash-photolysis multipass cell coupled with dual-comb spectroscopy in the short-wave (near 2.9 μm) and long-wave (near 7.9 or 8.9 μm) mid-IR regions to quantitatively measure the reaction species before and after laser photolysis. HOOH is produced via heating of urea-hydrogen peroxide. This is an excellent piece of work and suitable for JPC Letters.

Major advances reported: The authors measure k(T) for OH + HOO using direct detection of reactant precursor (HOOH) and radical species of title reaction (OH and HOO). The current paper reaffirms previous measurements from Assaf et al. (which involved one of the senior authors of this paper, CF). Through detailed kinetics analysis, the rate coefficient for OH + HOOH is also reported and is consistent with the literature (a discrepancy of 16% is noted, but this is a reasonable level of uncertainty). The authors also conclude there is no impact of water concentration on the rate coefficient for OH + HOO. A convincing plot of the current data compared with all measurements in the literature (Figure 5) is provided.

Immediate significance: This work reconciles a disparity in literature ascribed to issues in definition of initial conditions of reactant concentrations based mostly on the direct quantification of HOOH via rotationally resolved IR-absorption spectra. The rate coefficient for this reaction is important to combustion and atmospheric chemistry.

Technical suggestions: Although outside the scope of work, a welcomed addition to this paper would be an examination of the influence of different colliders – ranging from He to carbon dioxide to assess the role, if any, that collision efficiency might play in the reported rate coefficients.

<Response & Revision 1-1>

We thank the reviewer for this suggestion. For the previous experiments carried out by Assaf et al. (reference 6), ~50 Torr He was used as the bath gas in their experiments. Since the rate coefficient k_{OH+HO2} obtained in this work using ~30 Torr Ne as the bath gas is in excellent agreement with the value reported by Assaf *et al.*, the collision efficiency would not influence in the reported rate coefficients by using different colliders (He and N_2). According to other experiments (Table S2), the rate coefficient k_{OH+HO2} is not sensitive to the total pressure, indicating the collision efficiency might not play on the rate coefficients.

Also, no comparison is made to theoretical treatment of this reaction, such as Burke et al., Proc. Combust. Inst., 2013. Why not? I realize this is an experimental-focused effort to resolve experimental discrepancies, which it certainly accomplishes, but some comments on such a comparison, even if in the SM, would be useful.

<Response & Revision 1-2>

We thank the reviewer for this suggestion. We have added a brief description of the experimental and theoretical studies of the reaction OH + HO_2 in Note S1. A comparison of the experimental and theoretical results of the k_{OH+HO2} from previous studies at around 300 K is shown in Figure S1.

Reviewer: 2

Comments:

The work described in this manuscript is motivated by a clear discrepancy (a factor of 10) between measured values of the rate coefficient at room temperature for the reaction of two radicals, OH and HO2. As both radicals play important roles in atmospheric chemistry and their cross reactions play a significant role in their removal from the upper troposphere, it is particularly important that the discrepancy be resolved.

The manuscript describes a beautiful collaboration between excellent investigators located nearly half a world apart. They apply a new experimental approach, time-resolved dual-comb spectroscopy, that for the first time directly measures the concentration of each radical spectroscopically. Moreover, the result clearly confirms the value of the rate coefficient reported previously by reference 6, contradicting the more recent report in reference 7. In addition, these experiments investigated the reported enhancement of the OH + HO2 reaction by water as reported in reference 7, but the present work did not confirm the previous results.

My only significant criticism of the manuscript is that the description of the reaction mechanism is rather confusing and overly brief. Essentially the authors try to do that using only the first half of the last sentence on page 5 that continues onto page 6. I puzzled over this description until I found Table S1 in the supplemental material that contains the kinetic model and the rate coefficients. I recommend moving this table to the text and using it and two or three well-crafted sentences to clearly describe this most important information.

<Response & Revision 2-1>

We thank the reviewer for this suggestion. We have rewritten this part of the content in the revised manuscript. The table of the kinetic model has moved to the main text.

There are a couple of additional items that I would like to see included in the text.

1. It appears (see figure 3) that the 248 nanometer photolysis of H2O2 produces only 2OH and no H + HO2 directly. Is that correct?

<Response & Revision 2-2>

The photodissociation of H_2O_2 at 248 nm has been well evaluated in previous experiments (J. Chem. Phys. 1990, 92, 996 and J. Chem. Phys. 2007, 126, 186101). The quantum yields of OH, O + H_2O, and H + HO_2 were obtained to be ~2, <0.02, and <0.01, respectively. We have added a description of the photodissociation of H_2O_2 at 248 nm in the main text.

2. The concentration of the radicals were measured using their absorption spectrum. The particular lines, and the values of the absorption coefficients used, should be given in the text. This information would be helpful for anyone who wanted to repeat the experiment and would give all readers an idea of the relative accuracy of the concentration measurements of each radical.

<Response & Revision 2-3>

We thank the reviewer for this suggestion. We have added the line strengths for the measured OH and HO_2 transitions in the main text.

Reviewer: 3

Comments:

The authors present a study of the OH + HO2 reaction, and compare it with previous studies of the same reaction, in an attempt to consolidate findings and solidify a rate coefficient to be used for this reaction. The use of frequency comb spectroscopy is beneficial here, in that both HO2 and OH can be monitored. There are a few questions I think should be addressed in either the main manuscript or in the SI:

1. The use of the urea-H2O2 precursor: what role does the SiO2 play in the precursor preparation? Have there been previous studies on the thermal decomposition of the SiO2/urea-H2O2 mix that show H2O2 is the only gaseous compound evolved at this temperature? There should be a discussion or citation here to showcase that any thermal decomposition products (that could interfere with the OH + HO2 reaction) have been considered and discounted.

<Response & Revision 3-1>

We thank the reviewer for this suggestion. The thermal decomposition of the H_2O_2−urea complex has been adopted in various experiments for generation of stable and gaseous H_2O_2 (J. Phys. Chem. A 2006, 110, 3330 and J. Phys. Chem. A 2010, 114, 5718). In this work, by monitoring the precursor mixtures with both UV and IR spectroscopy, we confirmed that the

pure H_2O_2 can be produced without other impurities such as H_2O, HNO_3, and HNCO from the H_2O_2–urea/SiO_2 mixture after 30 minutes warming up and during stable heating at below 50 °C. We have added the citations and description in the main text.

2. The concentration of OH relies on the measurements from the author's previous paper of line strengths. I have a few questions regarding this:

a) For the concentration of OH, the author's previous paper and this one relies on the assumption that the quantum yield of OH from 248 nm photolysis of H2O2 is 2. This is the recommended value, but the study from Nesbitt and coworkers (https://doi.org/10.1063/1.464735) does show a power dependence to the quantum yield, which shifts away from 2 as the power is increased. The authors have looked at multiple 248 nm laser powers -- do they see any power dependence to the quantum yield of OH?

<Response & Revision 3-2>

The photodissociation of H_2O_2 might proceed at 248 nm via three different primary processes:

$$H_2O_2 + h\nu \rightarrow 2OH \qquad \lambda<557 \text{ nm} \qquad (1)$$
$$\rightarrow O + H_2O \qquad \lambda<359 \text{ nm} \qquad (2)$$
$$\rightarrow H + HO_2 \qquad \lambda<324 \text{ nm} \qquad (3)$$

The quantum yields of the three channels has also been well evaluated in previous experiments by Ravishankara *et al.* (J. Chem. Phys. 1990, 92, 996) and Fittschen *et al.* (J. Chem. Phys. 2007, 126, 186101). Ravishankara *et al.* obtained that the quantum yield (Φ_1) of OH is 2.09 ± 0.36 and the quantum yields for O and H are $\Phi_2<0.002$ and $\Phi_3<0.0002$, respectively. Furthermore, Fittschen *et al.* also confirmed that the quantum yield for pathways (2) and (3) are $\Phi_2<0.02$ and $\Phi_3<0.01$, respectively. Both experiments employed the sensitive methods to evaluate the formation yield of O and H from the photodissociation of H_2O_2 at 248 nm. In addition, Ravishankara *et al.* also employed the pulsed photolysis laser-induced fluorescence apparatus to study OH quantum yields of the photodissociation of H_2O_2 at 248 nm and 222 nm (J. Chem. Phys. 1990, 92, 996 and J. Chem. Phys. 1992, 96, 5878). In the experiments of Ravishankara *et al.,* the relatively low photolysis energy of 0.1~3.3 mJ cm^{-2} was used. By comparison, Fittschen *et al.* studied the photodissociation of H_2O_2 using the photolysis laser with a pulse energy of 40 mJ cm^{-2} at 248 nm. Both experiments suggested that the OH quantum yield in 248 nm photolysis is 2. In comparison to above experiments, Nesbitt and coworkers determined the OH quantum yield by measuring the absorption spectra of the OH $X^2\Pi_{3/2}$ (1 ← 0) P(2.5) transitions and obtained the $\Phi_1 = 1.58\pm0.23$ at the experimental conditions with the photolysis energy of <37 mJ cm^{-2}. The absolute OH concentrations were obtained via Beer's law and the integrated transition cross sections which were determined in their previous work (J. Chem. Phys. 1987, 90, 5455). Because the integrated transition cross sections were obtained by analyzing the OH yields in the laser photolysis of HNO_3 and calculations of dipole moment function, transition cross sections of OH lines might have a large uncertainty and it might cause

that the OH concentrations were underestimated in the experiment of photodissociation of H_2O_2 at 248 nm. On the other hand, for Nesbitt's experiments, if considering that $\Phi_1 = 2$, the used absorption cross sections of OH line should be lower by a factor of ~1.3, and thus the corrected line strengths (or the Einstein A coefficients) of OH would be ~17% lower than the values reported by HITRAN, which is comparable well with our current finding that the OH line strengths are ~25% lower compared to that reported by HITRAN. Moreover, in our previous experiments on the measurements of OH line strengths and rate coefficient of the reaction OH + H_2O, we employed the photolysis laser with the energy of 19~42.8 mJ cm^{-2}, but we did not observe any power dependence to the quantum yield of OH.

b) The previous paper showed a consistent 25% disagreement of the line strength compared to that reported by HITRAN for the OH transition used here. If the authors used the line strengths reported by HITRAN instead of the ones derived in the previous paper, does that make a difference to the derived reaction rate coefficient?

<Response & Revision 3-3>

If the OH concentrations were estimated using the line strengths from HITRAN, the kinetic simulated curves cannot fit well with the measured concentration profiles of both OH and HO_2, as shown in Figure R1. According to the kinetic simulations (Figure R2), the yields of HO_2 are strongly sensitive to the value of k_{OH+HO2} and the influence of the OH+HO_2 reaction to the HO_2 yields will become larger while the radical concentrations are increased. Therefore, it would be important to fit the simulation curves with the time traces of OH and HO_2 measured under conditions with different initial concentrations of OH. In addition, the line strengths of HO_2 were also measured by simultaneous analysis of high-resolution time-resolved spectra of HCl and HO_2 in the Cl + CH_3OH reaction system. Our determined HO_2 line strengths are higher than that from HITRAN by a factor of ~2.75, which is comparable to another experimental results (J. Phys. Chem. A, 2012, 116, 215). The absolute line strengths listed in current HITRAN database for both OH and HO_2 would need to be revised and updated. We have given the line strengths of measured OH and HO_2 lines in the main text for anyone who wants to repeat the experiment or to use for estimation of radical concentrations.

With the kinetic simulations, the 5% error of the measured $k_{OH+H2O2}$ could cause the uncertainty of 5% on the determination of k_{OH+HO2}. The influences on k_{OH+HO2} caused by the possible errors of the self-reactions of OH and HO_2 (30%) were also considered and estimated less than 1%. In addition, by simultaneous analysis of the OH and HO_2 time traces obtained under different experimental conditions, the uncertainty of k_{OH+HO2} caused by the errors of the line strengths of OH (7%) and HO_2 (4%), spectral analysis (4%), and the effective absorption path (7%) was evaluated to be ~9%. Considering all possible errors, the overall uncertainty of the k_{OH+HO2} was hence estimated to be ~11% in this work.

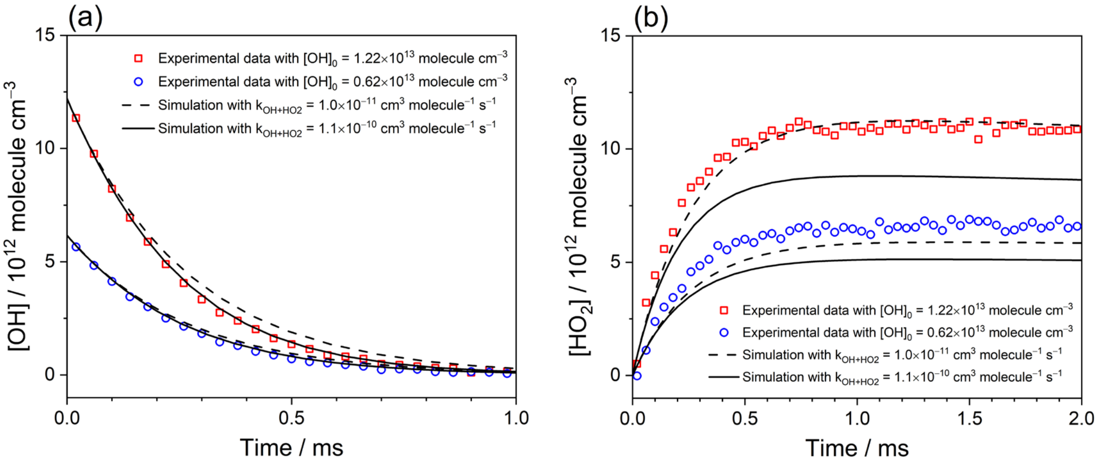

Figure R1. Comparison of the measured time traces and the simulated profiles of OH and HO_2 at different conditions. The concentration temporal profiles of (a) OH and (b) HO_2 were recorded after the 248 nm irradiation of the flowing mixture of H_2O_2/N_2 ($[H_2O_2]_0 \approx 1.9\times10^{15}$ molecules cm^{-3}, $P_T \approx 30.3$ Torr, 296 K) with photolysis energies of 38.0 (red) and 19.0 (blue) mJ cm^{-2}. The data correspond to experiment set 1 and 3 listed in Table S1, but the OH concentration profiles were obtained by employing the line intensity of OH $X^2\Pi_{3/2}$ (1 ← 0) P(4.5)f transition with the value from Hitran database (S = 4.55×10^{-20} cm molecule^{-1}). The solid curves and the dash lines represent the simulated profiles derived using the kinetic model, as shown in Table 1, with $k_{OH+HO2} = 1.1\times10^{-10}$ and 1.0×10^{-11} cm^3 molecule^{-1} s^{-1}, respectively.

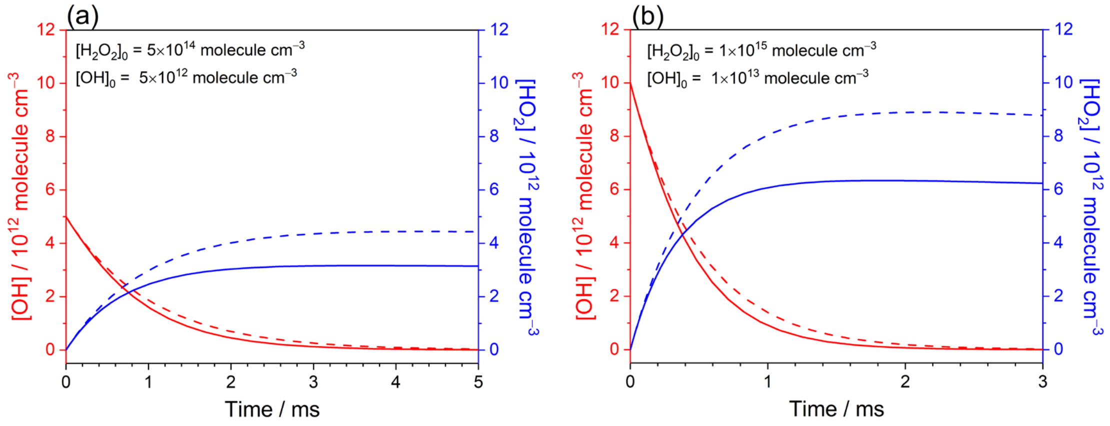

Figure R2. Comparison of the simulated temporal profiles of OH and HO_2 with different radical concentrations. (a) $[H_2O_2]_0 = 5\times10^{14}$ molecule cm^{-3} and $[OH]_0 = 5\times10^{12}$ molecule cm^{-3}. (b) $[H_2O_2]_0 = 1\times10^{15}$ molecule cm^{-3} and $[OH]_0 = 1\times10^{13}$ molecule cm^{-3}. The solid curves and the dash lines represent the simulated profiles derived using the kinetic model, as shown in Table 1, with $k_{OH+HO2} = 1.1\times10^{-10}$ and 1.0×10^{-11} cm^3 molecule^{-1} s^{-1}, respectively.

3. It reads as though the authors used only one transition for OH to derive the final reaction rate coefficient (bottom of page 10). Is this correct? How sensitive is the measurement to which transition is chosen to be used? Do the authors use multiple transitions for HO2, or just a single one? It seems that the power of a frequency comb spectrometer lies in being able to use a combined fit to multiple transitions to get a more accurate representation of the kinetics, so I'm curious if the authors took advantage of it.

<Response & Revision 3-4>

To avoid the interference from strong absorption lines of H_2O, H_2O_2 as well as HO_2, the spectral region near 3407 cm^{-1} was hence chosen to monitor the OH radicals. The OH $X^2\Pi_{3/2}$ (1 ← 0) P(4.5) transitions near 3407 cm^{-1} also have the relatively strong line intensity. Because a small HO_2 absorption signal was observed and it overlaps with the OH line near 3408 cm^{-1}, we mainly analyzed the temporal profiles of OH by monitoring the absorption line of OH at 3407.612 cm^{-1}. We also used other OH lines near 3484 cm^{-1} to measure the time traces of OH for the experiments without addition of water. Figure S5 shows the comparison of the measured and simulated time traces of OH and HO_2 at varied conditions. Here, the OH time traces were obtained by analyzing the time-resolved spectra of the OH $X^2\Pi_{3/2}$ (1 ← 0) P(2.5) transitions near 3484 cm^{-1} and the HO_2 time traces were obtained by analyzing the time-resolved spectra of five absorption peaks near 1123 cm^{-1}. The final results for determining the k_{OH+HO2} are consistent as the experiments shown in the main text. For the experiments with addition of water, because there are some absorption lines of H_2O near 3484 cm^{-1}, it is not suitable for using this spectral range to probe OH under the condition in the presence of high water concentration. We have added a detailed description in the main text and the caption of Figure 3. The line strength of the OH $X^2\Pi_{3/2}$ (1 ← 0) P(4.5)f transition was determined to be $(3.38 \pm 0.25)\times10^{-20}$ cm $molecule^{-1}$ in our previous work. By probing this OH transition, the detection limit of OH down to $\sim1 \times10^{11}$ molecule cm^{-3} can be achieved at the time resolution of tens of μs. In time-resolved dual-comb spectroscopy, a trade-off exists among the spectral resolution, temporal resolution, and spectral coverage of the frequency combs. Currently, the single-shot spectral coverage of each dual-comb spectrometer is only 1~2 cm^{-1} for achieving the spectral measurements with both high spectral and temporal resolutions. Although the laser has a wide tunable range, we cannot measure time-resolved spectra covering many OH ro-vibrational lines with different *J* numbers in the same time. In this work, we have chosen the best approach to carry out the experiments for the investigation on the k_{OH+HO2}.

4. If I recall correctly, OH is produced vibrationally cold but rotationally hot from 248 nm photolysis of H2O2. How is rotational relaxation accounted for in your kinetic analysis? Can you see evidence of any rotational relaxation based on multiple rovibrational transitions being

probed? I believe rotational energy transfer rates for OH are on the order of 10^-10 cm3 molecule−1 s−1 (at least for helium, see https://iopscience.iop.org/article/10.1088/1361-6463/aa5b28/meta and references therein, such as reference 3), so perhaps the rotational relaxation is fast under your pressure conditions and you observe a thermalized distribution? Are you able to fit the rotational temperature of your observed OH?

<Response & Revision 3-5>

According to the reference (J. Phys. D: Appl. Phys., 2017, 50 114003), the vibrational relaxation rate coefficient for OH with the collider of N_2 is ~2.1×10^{-10} cm^3 s^{-1}. Therefore, the vibrational relaxation time of OH in our system with ~30 Torr N_2 is only ~5 ns, which is much shorter than the used time resolution (40 μs). In this experiment, the OH was probed under thermal equilibrium. To obtain accurate rotational temperature of OH, it is required to measure time-resolved spectra covering many OH ro-vibrational lines with different *J* numbers in the same time. For our current experimental approach, it would be tricky to achieve.

5. As with question 1 above, some additional experimental detail would be appreciated, perhaps in the SI, or at least reference to the appropriate papers. For example, what is the residence time in the kinetic gas cell, and are there any issues around wall-loss (not included in the kinetic model)? Have the authors noticed a change in any of the measurements if they do not irradiate the H2O2 precursor in the UV absorption cell prior to its entrance into the gas cell used for kinetics (what is the D2 lamp make/model and its flux)?

<Response & Revision 3-6>

We thank the reviewer for pointing this out to us. We have added the experimental detail in the main text and the caption of Figure S2. The mixing ratio of the H_2O_2/N_2 pre-mixtures before injection into the multi-pass cell was determined by employing the measured UV absorption spectra and the absorption cross section of H_2O_2 in region 215–220 nm. Before entering the multipass cell, the generated water-free H_2O_2 was first carried to a UV absorption cell by a well-calibrated nitrogen flow at 1000 sccm. A D_2 lamp (StellarNet Inc., SL3) with an average power of 0.24 mW in the range of 190-280 nm was sent to the UV absorption cell and detected by a UV spectrometer (Oceanhood, XS11639) to evaluate the concentration of H_2O_2 pre-mixtures. The photodissociation of H_2O_2 by the D_2 lamp in the UV absorption cell was estimated to be <0.003%, which can be negligible in the experiment. In addition, the initial concentration of H_2O_2 in the multipass cell was also measured by using infrared absorption spectroscopy for double confirming that no loss of H_2O_2 was caused in the flowing system. In the multipass cell, the mirrors and the windows of the cell were purged by streams of nitrogen, which gave rise to a total flow rate of ~1050 sccm and a residence time around 3 s. Because the reactions involving OH were relatively fast, the OH could be reacted away within 1~2 ms, we did not consider that the wall-loss of radicals could be an issue.

Minor points:

1. Please remove the hyphen between the number and its unit for 248 nm.
2. "Summary" is misspelled in the caption for Figure 5.

<Response & Revision 3-7>

We thank the reviewer for pointing this out to us. We have revised it accordingly.

Reviewer: 4

Comments:

The paper by Chen et al is an experimental study, aimed at addressing a discrepancy in recent measurements of the OH + HO2 reaction rate coefficient. OH is produces by laser flash photolysis of H2O2/N2 mixtures in a flow cell; subsequent reaction of OH + H2O2 forms HO2, and the time-resolved yield of HO2 is sensitive to the reaction of OH + HO2. Both OH and HO2 are probed by multi-pass dual-frequency comb spectrometers in the mid-IR and far IR range. Quantification of OH and HO2 is possible via earlier vibrational line strength determinations by the same group. Recorded time traces at several H2O2 and initial radical concentrations are compared to kinetic model predictions, and from this the value for k(OH+HO2) is derived. The study is carefully conducted and clearly written. The major finding of the paper is that the rate coefficient k(OH+HO2) = (1.1+/-0.14)e-10 cm3/molecules.

From a technical standpoint, this work represents state-of-the-art current capability of time-resolved frequency-comb probing of reactive radical species. However, the main scientific result is not a transformative new finding but rather a confirmation of the earlier value by one of the authors for a single rate coefficient of a single reaction (albeit an important one). Furthermore, the result agrees quantitatively with the current IUPAC recommendation. Therefore, although the present result increases our confidence in the rate coefficient for OH + HO2, it is not likely to impact modeling efforts.

Overall, this is a thorough and careful study that does not report an urgent new development and therefore would better fit into another journal like JPCA or IJCK. In addition, I would suggest several other points to assist the reader:

<Response & Revision 4-1>

The important of this work is to resolve the large discrepancy of the rate coefficient for OH + HO_2 obtained from both recent experimental and theoretical results, as shown in Figure S1. In the most recent experiment (J. Am. Chem. Soc. Au, 2023, 3, 1684), Speak *et al.* reported a much slower rate coefficient (~1×10^{-11} cm^3 $molecule^{-1}$ s^{-1}) and proposed that the rate coefficients have a significant enhancement under the conditions in the presence of water. However, in this work, the k_{OH+HO2} was determined to be 1.1×10^{-10} cm^3 $molecule^{-1}$ s^{-1} and no enhancement in the rate coefficient can be observed, indicating an obvious disagreement with

the results reported by Speak *et al.* Therefore, this work as an independent experiment that was implemented based on direct quantitation of all key reactants and carefully kinetic evaluations would be critical and essential in revisiting the rate coefficient of the reaction between OH and HO_2.

- On pages 5 – 6 the authors refer to a 2023 study by Chang et al (ref. 19) in which OH line absorption line strengths are quantified by referencing them to H2O2. The setup in that study seems very similar to this one, including the method of OH production and the probe method, dual-comb spectroscopy. What are the difference in the two studies, that make the previous study insensitive to OH + HO2 but the present one sensitive to this reaction?

<Response & Revision 4-2>

According to the kinetic simulations, the yields of HO_2 are strongly sensitive to the value of k_{OH+HO2} and the influence of the $OH+HO_2$ reaction to the HO_2 yields will become larger while the radical concentrations are increased, as shown in Figure R2. Therefore, in our previous work, the relatively lower OH concentrations were employed for studying the line strengths of OH and the kinetics of the $OH+H_2O_2$ reaction. In comparison to the previous experiments, herein we used higher initial concentrations of OH to better evaluate the k_{OH+HO2} based on the measured concentration time traces of both OH and HO_2.

- Please include a table of experimental conditions, including the initial radical concentrations (probably in the SI). Please consider reporting the reactant concentrations as number densities rather than partial pressures in Torr – it makes estimating reaction rates easier.

<Response & Revision 4-3>

We thank the reviewer for pointing this out to us. We have revised it accordingly.

- Are the present results exclusively sensitive to OH + HO2, or could correlation with some other reactions significantly influence the determination of k(OH+HO2)? The authors should consider a sensitivity analysis using any available chemical modeling software like Chemkin or Cantera, in order to support the idea that the present results are mainly or exclusively sensitive to the reaction of interest.

<Response & Revision 4-4>

In this work, we used the relatively simple and clear kinetic model, as shown in Table 1. Most importantly, all key parameters such as the rate coefficient of the reaction OH + H_2O_2 and absolute line strengths of H_2O_2, OH, and HO_2 were also measured using the same experimental system to reduce the systematic errors and to avoid unknown uncertainty. With the kinetic

simulations, the 5% error of the measured $k_{OH+H2O2}$ could cause the uncertainty of 5% on the determination of k_{OH+HO2}. The influences on k_{OH+HO2} caused by the possible errors of the self-reactions of OH and HO_2 (30%) were also considered and estimated less than 1%. In addition, by simultaneous analysis of the OH and HO_2 time traces obtained under different experimental conditions, the uncertainty of k_{OH+HO2} caused by the errors of the line strengths of OH (7%) and HO_2 (4%), spectral analysis (4%), and the effective absorption path (7%) was evaluated to be ~9%. Considering all possible errors, the overall uncertainty of the k_{OH+HO2} was hence estimated to be ~11% in this work.

- The experiment includes a UV spectrometer to measure [H2O2]. Do the IR dual-comb measurements of pre-photolysis [H2O2] agree with the UV measurements? Those are not mentioned in the paper.

<Response & Revision 4-5>

We thank the reviewer for pointing this out to us. We used both UV and IR spectroscopy to measure the pre-photolysis $[H_2O_2]_0$ for double confirming that no loss of H_2O_2 was caused in the flowing system. We have added the clear description of the preparation and determination of H_2O_2 in the main text.

- Is it possible to obtain time traces of H2O2 and H2O (under water-free conditions) from these measurements? Several reactions in the mechanism make H2O2 and H2O as products, and it would be interesting to compare those experimental results to model predictions, if it were possible.

<Response & Revision 4-6>

It would be difficult to obtain time traces with good SNR for the H_2O with low concentrations (~10^{13} molecule cm^{-3}) generated from the reactions, because the infrared comb laser would be absorbed and affected by the water in atmosphere before introducing into the reactor. For time traces of H_2O_2, we can observe the depletion of H_2O_2 caused by flash photolysis and the reaction of OH + H_2O_2, but it would be difficult to evaluate the yields of H_2O_2 formed from the self-reaction of OH (R4b) and HO_2 (R5), because the reaction rates of these reactions are too slow (<40 s^{-1}) under the employed experimental conditions.
